# Alpha-Interferon Suppresses Hepadnavirus Transcription by Altering Epigenetic Modification of cccDNA Minichromosomes

**DOI:** 10.1371/journal.ppat.1003613

**Published:** 2013-09-12

**Authors:** Fei Liu, Matthew Campagna, Yonghe Qi, Xuesen Zhao, Fang Guo, Chunxiao Xu, Sichen Li, Wenhui Li, Timothy M. Block, Jinhong Chang, Ju-Tao Guo

**Affiliations:** 1 Drexel Institute for Biotechnology and Virology Research, Department of Microbiology and Immunology, Drexel University College of Medicine, Doylestown, Pennsylvania, United States of America; 2 Institute for Hepatitis and Virology Research, Hepatitis B Foundation, Doylestown, Pennsylvania, United States of America; 3 National Institute of Biological Sciences, Beijing, People's Republic of China; 4 Neurology Department, David Geffen Medical School, University of Los Angeles, Los Angeles, California, United States of America; University of California, San Diego, United States of America

## Abstract

Covalently closed circular DNA (cccDNA) of hepadnaviruses exists as an episomal minichromosome in the nucleus of infected hepatocyte and serves as the transcriptional template for viral mRNA synthesis. Elimination of cccDNA is the prerequisite for either a therapeutic cure or immunological resolution of HBV infection. Although accumulating evidence suggests that inflammatory cytokines-mediated cure of virally infected hepatocytes does occur and plays an essential role in the resolution of an acute HBV infection, the molecular mechanism by which the cytokines eliminate cccDNA and/or suppress its transcription remains elusive. This is largely due to the lack of convenient cell culture systems supporting efficient HBV infection and cccDNA formation to allow detailed molecular analyses. In this study, we took the advantage of a chicken hepatoma cell line that supports tetracycline-inducible duck hepatitis B virus (DHBV) replication and established an experimental condition mimicking the virally infected hepatocytes in which DHBV pregenomic (pg) RNA transcription and DNA replication are solely dependent on cccDNA. This cell culture system allowed us to demonstrate that cccDNA transcription required histone deacetylase activity and IFN-α induced a profound and long-lasting suppression of cccDNA transcription, which required protein synthesis and was associated with the reduction of acetylated histone H3 lysine 9 (H3K9) and 27 (H3K27) in cccDNA minichromosomes. Moreover, IFN-α treatment also induced a delayed response that appeared to accelerate the decay of cccDNA. Our studies have thus shed light on the molecular mechanism by which IFN-α noncytolytically controls hepadnavirus infection.

## Author Summary

Hepatitis B virus (HBV) infection affects approximately one-third of the world population and more than 350 million people are chronically infected by the virus, for which the currently available antiviral therapies fail to provide a cure. This is because the HBV DNA polymerase inhibitors have no direct effect on the nuclear form of HBV genome, the covalently closed circular (ccc) DNA. Elimination or transcriptional silencing of cccDNA is the prerequisite for either a therapeutic cure or immunological resolution of HBV infection. However, due to the lack of proper experimental systems, the molecular mechanism of cccDNA biosynthesis, maintenance and transcription regulation remains to be elucidated. We report herein the establishment of a cell-based assay where the replication of duck hepatitis B virus (DHBV), a close relative of HBV, is supported by cccDNA. This experimental system not only allows us to demonstrate the unique property of alpha-interferon suppression of cccDNA transcription, but also shows for the first time that DHBV cccDNA transcription requires histone deacetylase activity. It is conceivable that the principles revealed by studying DHBV cccDNA metabolism and transcription regulation should provide valuable insight in HBV cccDNA biology and clues for the development of therapeutics to control chronic hepatitis B.

## Introduction

Hepatitis B virus (HBV) is the prototype member of the *Hepadnaviridae* family and contains a relaxed circular (rc) partially double stranded DNA (3.2 kb in length) genome [1]–[3]. Upon entry into a hepatocyte, the nucleocapsid delivers the genomic rcDNA into the nucleus, where the rcDNA is converted into a covalently closed circular (ccc) DNA. The cccDNA exists as an episomal minichromosome and serves as the template for the transcription of viral RNAs [4], [5]. Hepadnaviruses replicate their genomes *via* a protein-primed reverse transcription of pre-genomic (pg) RNA in the cytoplasmic nucleocapsids, which are subsequently enveloped upon synthesis of rcDNA and secreted out of cells as virions [6], [7]. During the early phase of infection, additional cccDNA are produced from newly synthesized cytoplasmic rcDNA through an intracellular amplification pathway [8], [9]. These two pathways culminate in the formation of a regulated steady-state population of 5 to 50 cccDNA molecules per infected hepatocyte [4], [10], [11].

Persistent infection of hepadnaviruses relies on the stable maintenance and proper function of a cccDNA pool in the nucleus of an infected hepatocyte as the source of viral RNAs. Not surprisingly, the metabolism and transcriptional activity of cccDNA are subjected to regulation by host pathophysiological cues. For example, although the cccDNA is apparently stable in stationary hepatocytes [12], the molecules can be non-cytolytically purged from infected hepatocytes during the resolution of an acute HBV infection in vivo, which is most likely due to the antiviral responses induced by gamma interferon (IFN-γ) and other inflammatory cytokines [13]–[15]. In support of this notion, alpha-interferon (IFN-α) and interleukin-6 have been shown to reduce the amounts of viral RNA transcribed from cccDNA in cultured hepatocytes or HBV-infected uPA-SCID mice *in vivo* [16] [17], [18]. Moreover, clinical studies suggest that cccDNA transcription activity is approximately 10-fold lower in HBeAg-negative patients than that in HBeAg-positive patients [19], [20], suggesting that host immunopathological factors regulate the activity of cccDNA transcription in vivo.

In addition, despite a profound reduction in its total amount, cccDNA becomes the dominant form of HBV DNA upon suppression of viral DNA synthesis by nucleoside analogue therapies [21]–[24]. Elimination of the residual cccDNA and/or silencing of its transcriptional activity are obligatory for a therapeutic cure of HBV infection, which has rarely achieved in patients receiving prolonged treatment of highly active viral DNA polymerase inhibitors [21], [25]–[27]. On the contrary, pegylated IFN-α (pegIFN-α) is effective in achieving sustained virologic response, defined as HBeAg seroconversion and/or hepatitis B virus (HBV) DNA levels below 20,000 copies/mL at 6 months after completion of the therapy, in only 30% of hepatitis e antigen (HBeAg)-positive and 40% of HBeAg-negative cases [28]–[30]. However, the pegIFN-α therapy does promote HBsAg clearance or seroconversion in a small, but significant fraction of treated patients [28]. The antiviral mechanism of IFN-α and the reasons for the differential therapeutic responses among the treated patients remain to be elucidated. Hence, understanding the molecular mechanism of cccDNA metabolism and transcription regulation by inflammatory cytokines should advance our knowledge of viral pathogenesis as well as facilitate the development of antiviral therapeutics to cure chronic hepatitis B.

However, due to the lack of convenient cell culture systems for efficient HBV infection [31], [32], the transcriptional regulation of hepadnaviruses was investigated primarily with hepatoma cells transfected with HBV reporter plasmids or transgenic mice harboring an integrated linear HBV genome in the host chromosomes [33]–[35]. It is most likely that certain features of cccDNA transcription regulation could not be recapitulated in these surrogate systems [36], [37]. As an alternative approach, Levrero and colleagues studied HBV transcription in the linear full-length HBV DNA-transfected human hepatoma cells, in which cccDNA were ostensibly formed by circularization of input linear HBV DNA [38], [39]. However, it is difficult to investigate cccDNA metabolism in such a transient transfection system. In this study, we took advantage of a chicken hepatoma cell line that supports tetracycline-inducible duck hepatitis B virus (DHBV) replication and established an experimental condition mimicking the virally infected hepatocytes in which DHBV pgRNA transcription and DNA replication are solely dependent on cccDNA. Unlike the transfected cells that cccDNA is derived from the input linear DNA, our assay allows to study the metabolism and transcription regulation of cccDNA synthesized from its authentic precursor, rcDNA in the cytoplasmic nucleocapsids.

Obviously, due to the lack of the x protein in avihepadnaviruses, which is essential for the establishment of woodchuck hepatitis virus infection in vivo and plays an important role in regulation of HBV cccDNA transcription in virally infected human hepatocytes [37], [39], [40], the cccDNA metabolism and transcription regulation of DHBV may differ in molecular details from that of mammalian hepadnaviruses. However, just as studying other replication steps of hepadnaviruses, such as nucleocapsid assembly, priming, reverse transcription and cccDNA formation [31], [41]–[43], the principles revealed by studying DHBV cccDNA metabolism and transcription regulation by inflammatory cytokines should provide valuable insight in HBV cccDNA biology and clues for the development of therapeutics to control chronic hepatitis B.

## Results

### DHBV cccDNA produced in the chicken hepatoma cells is transcriptionally active

**Figure 1:**
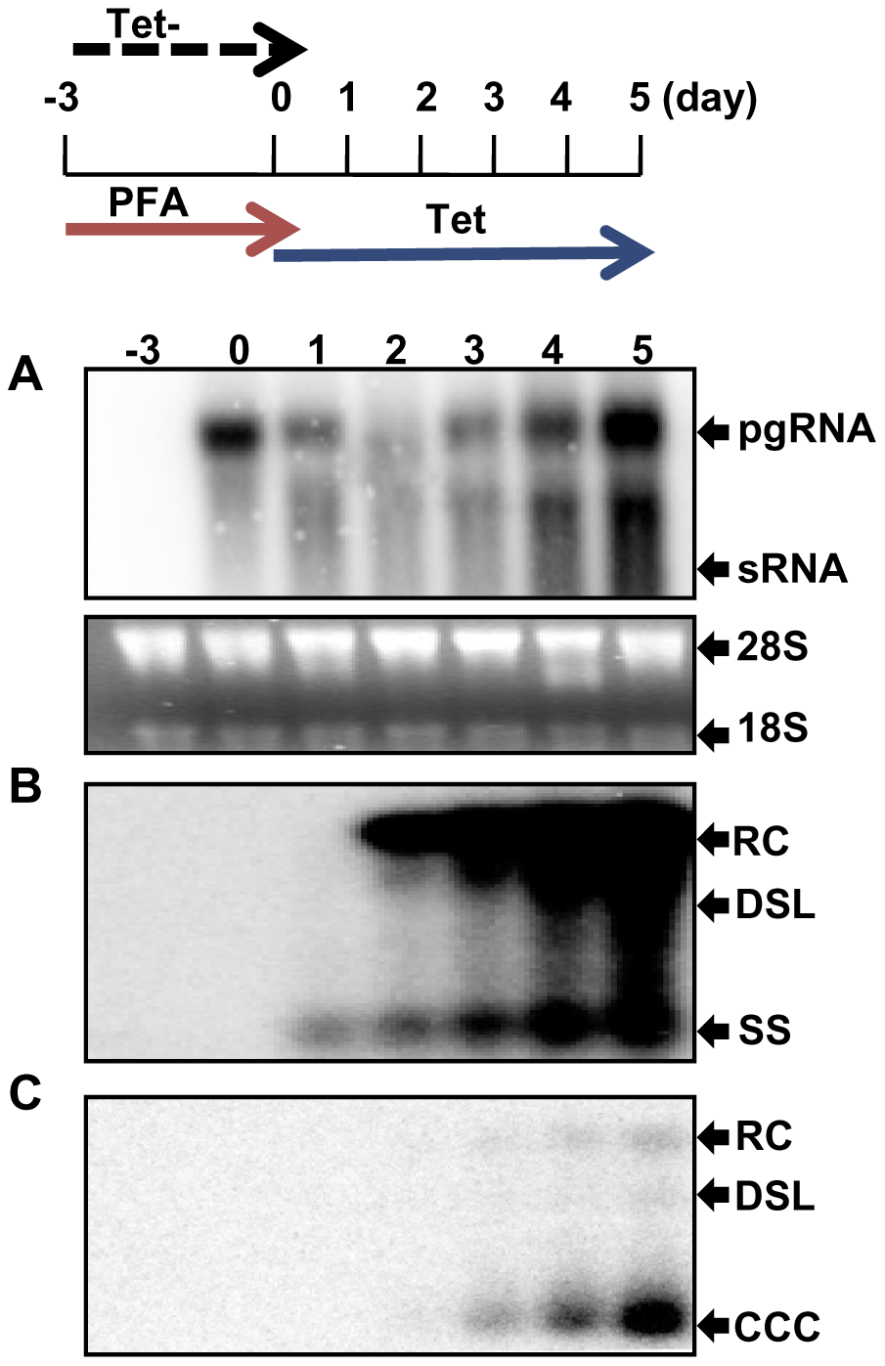
DHBV cccDNA molecules in dstet5 cells are transcriptionally active. Dstet5 cells were treated and harvested as depicted in the top panel. DHBV mRNA (A), cytoplasmic core DNA (B) and cccDNA (C) were determined by Northern and Southern blot hybridization, respectively. Ribosomal RNAs served as loading controls. pgRNA, pregenomic RNA; sRNA, mRNAs encoding envelope proteins; 28S and 18S, 28S and 18S rRNA, respectively; RC, relaxed circular DNA; DSL, double-stranded linear DNA; SS, single stranded DNA.

We previously established a chicken hepatoma-derived stable cell line harboring an integrated transgene for transcription of DHBV pgRNA in a tetracycline (tet) inducible manner, designated as dstet5 [44]. Upon removal of tet from culture medium, pgRNA was transcribed from the viral transgene integrated in the host cellular chromosome, which led to a sequential occurrence of viral protein translation, nucleocapsid assembly, DNA synthesis and cccDNA formation. To determine the transcriptional activity of the cccDNA, dstet5 cells were cultured in the absence of tet and presence of 2 mM foscarnet (PFA), a reversible DHBV DNA polymerase inhibitor, to arrest viral DNA synthesis [45]. Three days later, tet (1 µg/ml) was added back into culture medium to shut off pgRNA transcription from the transgene. Meanwhile, PFA was removed from the medium to resume the viral DNA synthesis in pgRNA-containing nucleocapsids and subsequent formation of cccDNA. As shown in Fig. 1, upon removal of PFA and addition of tet, the steady-state levels of DHBV pgRNA transcribed from the viral transgene declined quickly and reached the lowest point on day 2 (Fig. 1A). However, in parallel with the appearance and accumulation of core DNA since day 1 and cccDNA since day 3 (Figs. 1B and C), pgRNA gradually increased and reached the level comparable with that before the addition of tet into culture medium on day 5 (Fig. 1A). The results thus imply that the cccDNA synthesized in the chicken hepatoma cells are transcriptionally active.

### IFN-α potently inhibits the transcription of DHBV cccDNA minichromsomes

**Figure 2:**
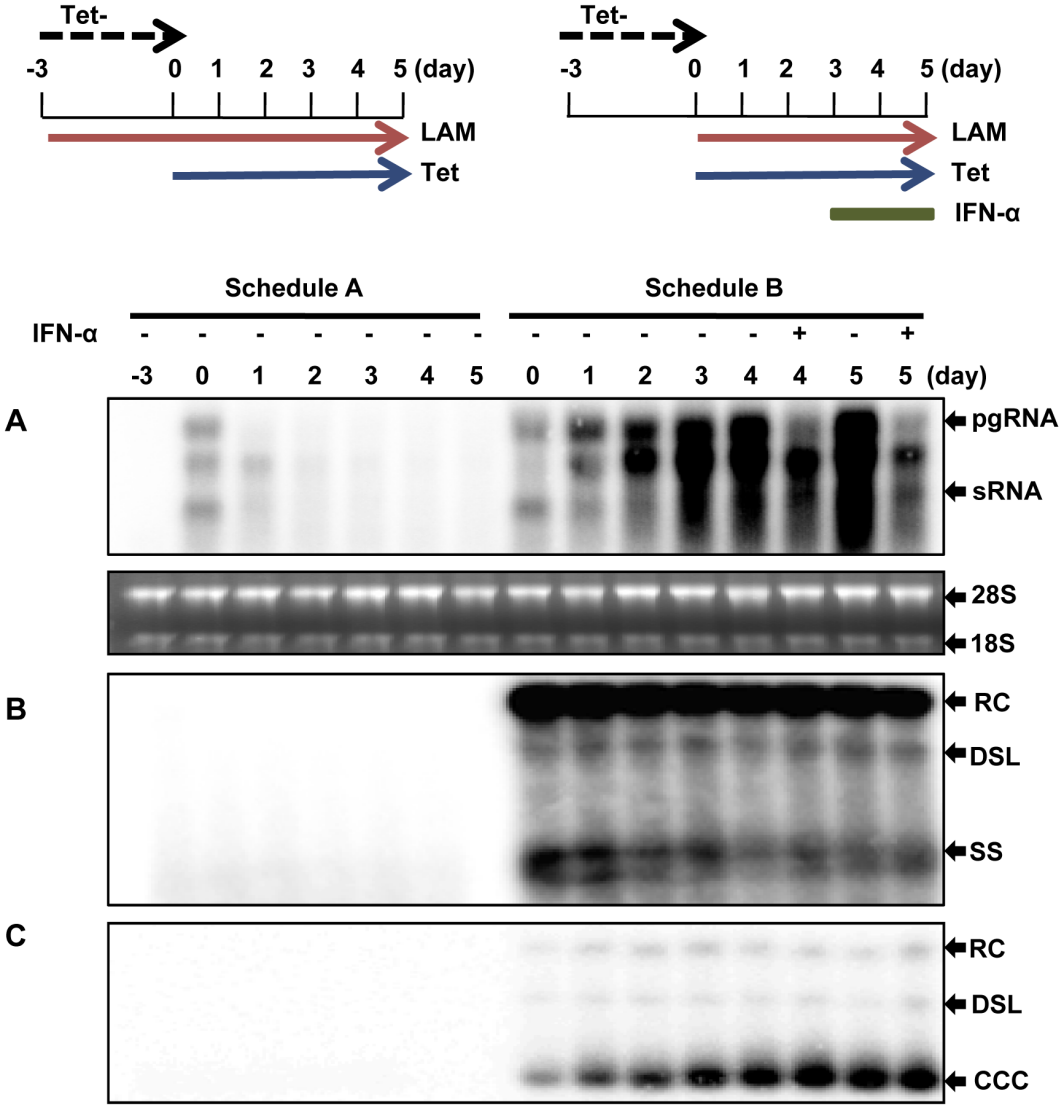
IFN-α reduces the amount of pgRNA transcribed from cccDNA. Dstet5 cells were treated and harvested as depicted in the top panel. DHBV mRNA (A), cytoplasmic core DNA (B) and cccDNA (C) were determined by Northern and Southern blot hybridization, respectively. Ribosomal RNAs served as loading controls. pgRNA, pregenomic RNA; sRNA, mRNAs encoding envelope proteins; 28S and 18S, 28S and 18S rRNA, respectively; RC, relaxed circular DNA; DSL, double-stranded linear DNA; SS, single stranded DNA.

The observed high transcriptional activity of DHBV cccDNA in dstet5 cells encouraged us to investigate if and how inflammatory cytokines regulate cccDNA transcription and whether or not the cccDNA minichromosome transcription is distinctly regulated and could thus be therapeutically targeted for selective inhibition of the viral gene expression. To achieve these goals, we first sought an appropriate experimental condition to study cccDNA transcription without interference from the integrated transgene. In fact, the tet-off inducible expression system allows us to promptly shut off the transgene transcription by simply adding tet into culture medium. Accordingly, we designed an experimental procedure where dstet5 cells were initially cultured in tet-free media to allow pgRNA transcription, DNA synthesis and cccDNA accumulation. Three days later, tet was added into the cultures to shut off transgene transcription. Meanwhile, lamivudine, an irreversible DHBV DNA polymerase inhibitor, was also added to arrest viral DNA synthesis and continuous cccDNA formation (Fig. 2, Treatment Schedule B). Because the pre-existing transgene-derived pgRNA were degraded with a half life of approximately 3 h [44] and became undetectable by the Northern blot hybridization assay at 24 h post addition of tet (Fig. 2A, Treatment Schedule A), the pgRNA detected after 24 h of tet addition ought to be primarily transcribed from cccDNA (Fig. 2A, Schedule B). Interestingly, a RNA species running slightly ahead of pgRNA was accumulated in cells treated with lamivudine and was apparently less sensitive to IFN-α treatment than pgRNA (Fig. 2A). Failure to remove the RNA species by micrococcal nuclease treatment of cell lysates suggests the RNA may be encapsidated in the nucleocapsids (data not shown). Hence, a plausible hypothesis is that the synthesis of minus strand DNA is not arrested by lamivudine at the priming stage, but at a site near the 3′ DR1 of pgRNA after the viral DNA polymerase translocation and elongation of minus stranded DNA synthesis. The observed RNA species was most likely the 5′ fragment of a pgRNA cleaved by RNase H. Studies are currently under way to further investigate this hypothesis.

Intriguingly, although lamivudine efficiently arrested DHBV DNA synthesis (Fig. 2B, Schedule A), it did not immediately stop cccDNA formation. The amount of cccDNA increased continuously for another three days since the addition of lamivudine, suggesting that inhibition of viral DNA synthesis did not prevent cccDNA formation from the pre-formed rcDNA (Fig. 2C, Schedule B). This is, in fact, consistent with a previous report that viral DNA polymerase activity was not required for cccDNA formation from rcDNA in virion particles [46]. Consequentially, although the transgene transcription was shut off and pre-existing pgRNA derived from the transgene were quickly degraded, the amount of DHBV pgRNA increased for at least three days after addition of tet, further suggesting that DHBV cccDNA molecules produced in the dstet5 cells were highly active in transcription. However, treatment of the cells with 100 U/ml chicken IFN-α, starting at 3 days after addition of tet and lamividine for 24 or 48 h, significantly reduced the amount of pgRNA, but not core DNA or cccDNA (Fig. 2A–C). Additional results presented in Fig. S1 further demonstrated that IFN-α treatment dose-dependently reduced pgRNA and was effective at a dose as low as 1 U/ml.

**Figure 3:**
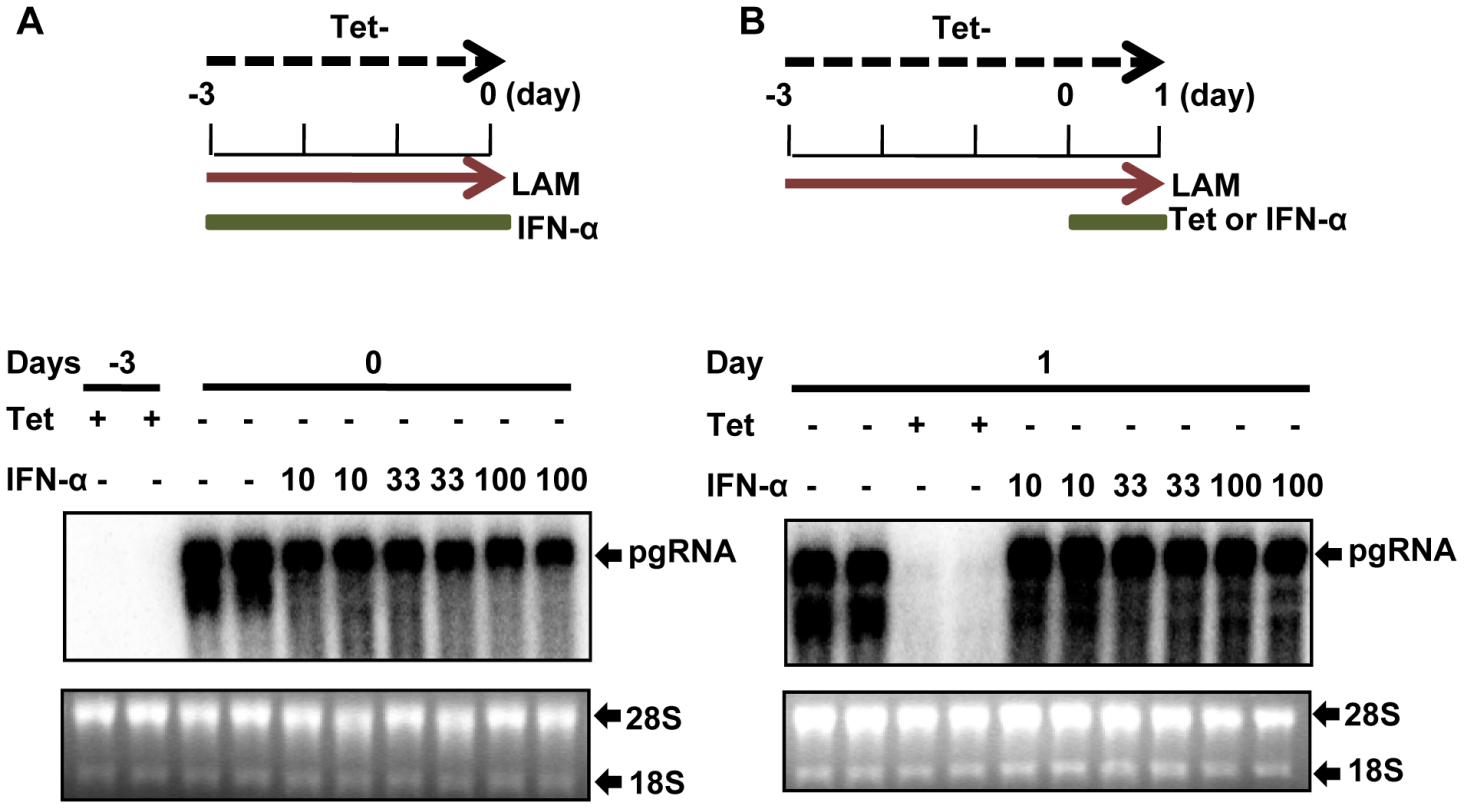
Effect of IFN-α on DHBV transgene transcription and pgRNA stability. (A) and (B) Dstet5 cells were treated and harvested as depicted in the top of each panel. DHBV mRNA was analyzed by Northern blot hybridization. Ribosomal RNAs served as loading controls. pgRNA, pregenomic RNA.

The observed reduction of viral RNA in IFN-α-treated dstet5 cells could be due to either inhibition of cccDNA transcription or accelerated post-transcriptional decay of viral RNA. Because the pgRNA derived from the transgene and cccDNA were identical in sequence, we thus determined whether IFN-α affected the stability of transgene-derived pgRNA. As demonstrated in Fig. 3A, IFN-α treatment of the dstet5 cells cultured in the absence of tet to allow pgRNA transcription from the transgene, but in the presence of 10 µM lamivudine to inhibit viral DNA synthesis and cccDNA formation for three days did not apparently alter the accumulation of pgRNA. In addition, when dstet5 cells were initially cultured in the absence of tet and presence of lamivudine for three days to allow the accumulation of pgRNA and followed by treatment with 100 U/ml IFN-α for 24 h, the cytokine also did not apparently alter the steady-state level of pgRNA. However, as expected, the levels of pgRNA were drastically reduced upon the addition of tet (Fig. 3B). Moreover, IFN-α treatment did not alter the decay kinetics of pgRNA upon shut-off of transgene transcription in dstet5 cells (Fig. S2). The results thus strongly indicated that IFN-α affected neither the transgene transcription nor the stability of pgRNA, but efficiently inhibited cccDNA transcription.

### IFN-α inhibition of DHBV cccDNA transcription requires protein synthesis

**Figure 4:**
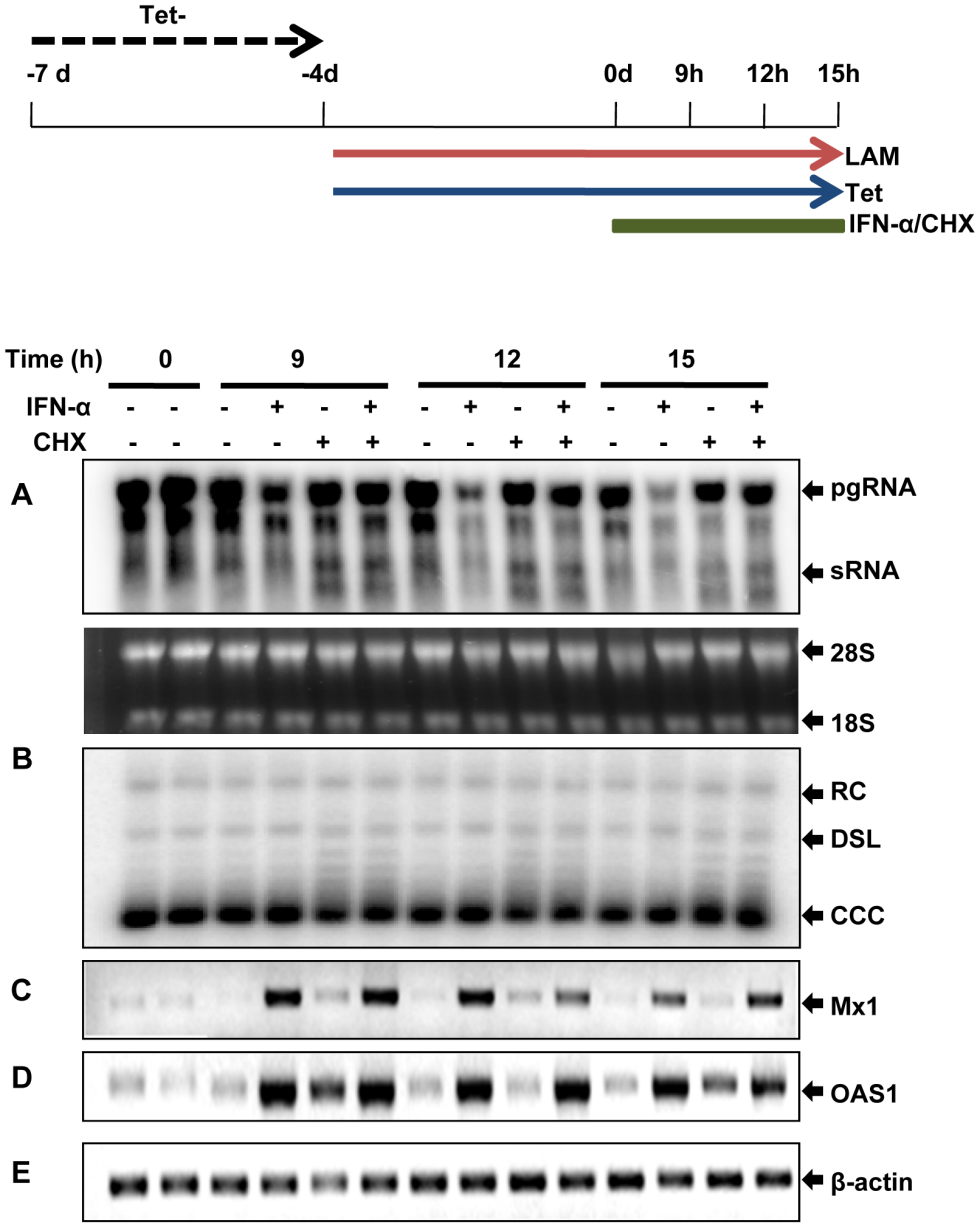
IFN-α inhibition of DHBV cccDNA transcription requires protein synthesis. Dstet5 cells were treated and harvested as depicted in the top panel. DHBV mRNA (A) and cccDNA (B) were determined by Northern and Southern blot hybridization, respectively. Ribosomal RNAs served as loading controls for the Northern blot. The levels of Mx1 (C), OAS1 (D) and β-actin (E) mRNA were determined by semi-quantitative RT-PCR assay. pgRNA, pregenomic RNA; sRNA, mRNAs encoding envelope proteins; 28S and 18S, 28S and 18S rRNA, respectively; RC, relaxed circular DNA; DSL, double-stranded linear DNA.

IFNs elicit an antiviral response by binding to their cognate receptors, which trigger a signaling cascade (JAK-STAT signaling pathway) leading to the expression of IFN-stimulated genes (ISGs), whose products exhibit antiviral activities [47]–[49]. Interestingly, the HBV genome contains a typical IFN-stimulated response element (ISRE), which was demonstrated recently to be essential for IFN-α inhibition of HBV cccDNA transcription in the unit-length linear HBV genome-transfected HepG2 cells, presumably through direct recruitment of STAT1 and STAT2 to the cccDNA minichromosomes [18]. However, a role of the putative ISRE in regulation of HBV transcription by IFN-α and interferon response factors 1 and 7 could not be revealed by another study [50]. Nevertheless, to investigate whether IFN-α inhibition of DHBV cccDNA transcription is mediated by the recruitment of pre-existing cellular proteins, such as STAT1/2, to the cccDNA minichromosomes or requires the synthesis of new antiviral protein(s), we tested the effect of cycloheximide (CHX), a protein translation inhibitor, on IFN-induced antiviral response on DHBV cccDNA transcription. As shown in Figs. 4 and S3, time course studies demonstrated that the reduction of pgRNA became evident as early as 9 h after the cytokine treatment. Interestingly, treatment of the cells with 10 µM of CHX completely abolished the inhibition of IFN-α on cccDNA transcription, but not the transcription of the two IFN-stimulated genes (ISGs), myxovirus resistance 1 (Mx1) and 2′-5′-oligoadenylate synthetase 1 (OAS1). The results thus imply that IFN-α inhibition of DHBV cccDNA transcription most likely requires induction of one or more cellular antiviral proteins, but the recruitment of STAT proteins and/or other pre-existing cellular proteins to the cccDNA minichromosomes might not be required or sufficient for the antiviral response.

### IFN-α induces a long-lasting suppression of DHBV replication

**Figure 5:**
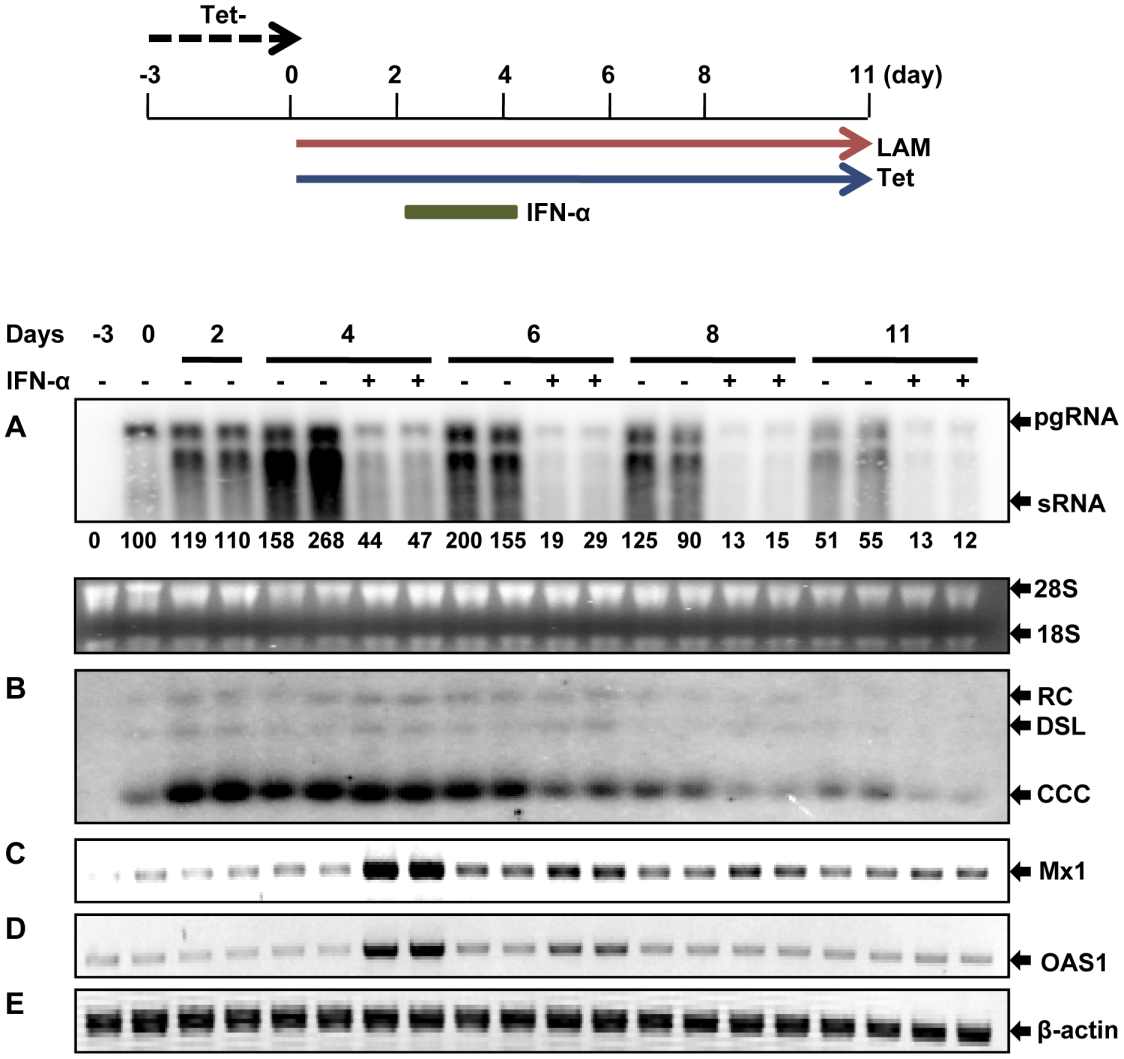
IFN-α induces a prolonged suppression of DHBV cccDNA transcription. Dstet5 cells were treated and harvested as depicted in the top panel. DHBV mRNA (A) and cccDNA (B) were determined by Northern and Southern blot hybridization, respectively. Ribosomal RNAs served as loading controls for the Northern blot hybridization. The amount of DHBV pgRNA was quantified by phosphoimager Quantity One (Bio-Rad) and presented as percentage of pgRNA on day 0 (panel A). The levels of Mx1 (C), OAS1 (D) and β-actin (E) mRNA were determined by semi-quantitative RT-PCR assay. pgRNA, pregenomic RNA; sRNA, mRNAs encoding envelope proteins; 28S and 18S, 28S and 18S rRNA, respectively; RC, relaxed circular DNA; DSL, double-stranded linear DNA.

Introduction of pegIFN-α into the clinics dramatically improved the antiviral efficacy of IFN-α therapy against chronic hepatitis B, primarily due to the prolonged half life of pegIFN-α over the standard IFN-α [28]. This clinical observation seems to indicate that the IFN-α-induced antiviral response might be short-lived and continuing engagement of the cytokine with its receptor is required for a persistent inhibition of HBV replication. To determine the longevity of IFN-α induced suppression on cccDNA transcription, dstet5 cells, upon cccDNA accumulation and shut-off of transgene expression as described above, were treated with IFN-α for two days and observed for additional 7 days after the cessation of the cytokine treatment. Consistent with the results presented above, two days of IFN-α treatment significantly reduced the amount of pgRNA, but not cccDNA (Figs. 5A and B). As expected, IFN-α induced the expression of Mx1 and OAS1 mRNA, which gradually decreased after the termination of the cytokine treatment (Figs. 5C and D). However, to our surprise, the level of pgRNA in IFN-α-treated cells continued declining after the treatment for at least 7 days (Fig. 5A). Furthermore, IFN-α appeared also to induce a delayed response that accelerated the decline of cccDNA, starting between 2 to 4 days after the treatment (Fig. 5B).

### Inhibition of cccDNA transcription is a primary mechanism of IFN-α against DHBV

**Figure 6:**
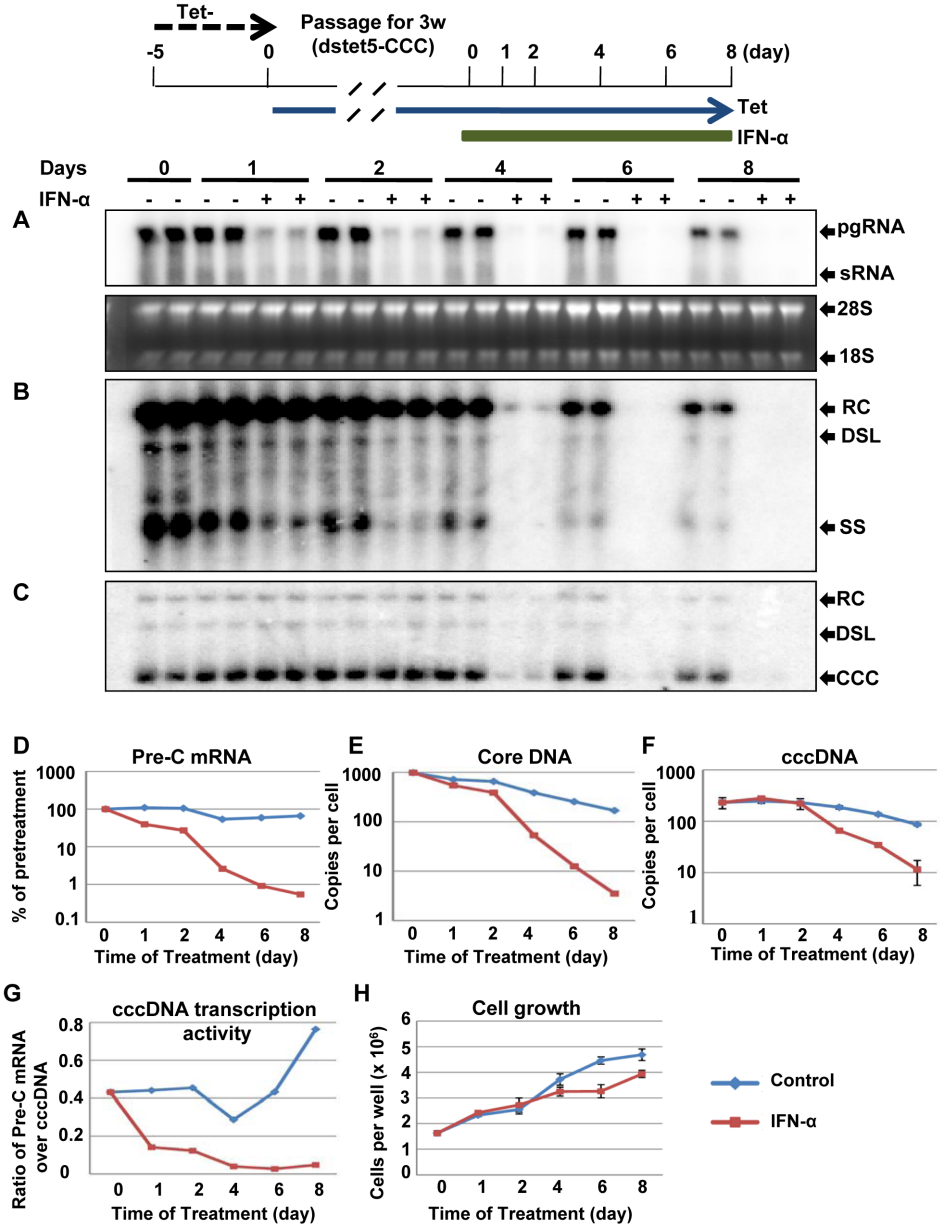
Decay kinetics of HBV replication intermediates in IFN-α treated cells. Dstet5 cells were treated and harvested as depicted in the top panel. DHBV mRNA (A), cytoplasmic core DNA (B) and cccDNA (C) were determined by Northern and Southern blot hybridization, respectively. Ribosomal RNAs served as loading controls for the Northern blot. (D) DHBV pre-C mRNA was quantified by a real-time RT-PCR assay and plotted as a ratio to β-actin mRNA. pgRNA, pregenomic RNA; sRNA, mRNAs encoding envelope proteins; 28S and 18S, 28S and 18S rRNA, respectively; RC, relaxed circular DNA; DSL, double-stranded linear DNA; SS, single stranded DNA. DHBV core DNA (E) and cccDNA (F) were quantified by qPCR assay and presented as copies per cell. (G) The molar ratios of pre-C mRNA over cccDNA were plotted to illustrate the cccDNA transcription activity. (H) The growth curves of dstet5 cells under the mock and IFN-α treatment. Mean values and standard derivations were calculated from a representative duplicated experiment.

In order to further investigate the pleiotropic effects of IFN-α on the DHBV replication cycle, dstet5 cells were initially cultured in the absence of tet for five days to allow the pool of cccDNA to be established. The cells were then cultured in the presence of tet to shut off the transgene transcription and passaged upon confluence for at least three weeks. As in DHBV persistently infected hepatocytes, the viral DNA replication cycle in these cells was only supported by cccDNA. As shown in Figs. 6A to C, although DHBV replication activity gradually declined in the untreated control cells as the cultures approached over-confluence, eight days of IFN-α treatment induced 121.5-, 48.2- and 7.6-fold net reduction of pre-C mRNA, core DNA and cccDNA, compared with the untreated controls, respectively (Figs. 6D–F). Because the CMV-tet promoter of DHBV transgene in the dstet5 cell line was designed to initiate transcription at the authentic initiation site of pgRNA, which precluded pre-C mRNA transcription [44], we thus preferred the quantification of pre-C mRNA that could only be transcribed from cccDNA.

Moreover, analyses of the decay kinetics of viral DNA replication intermediates revealed that a profound reduction of pgRNA or pre-C mRNA was observed within 24 h of IFN-α treatment (Figs. 6A and D). Reduction of single-stranded core DNA and rcDNA also became evident at 24 and 48 h of the treatment, respectively (Figs. 6B and E). In marked contrast, the amounts of cccDNA were slightly increased during the first two days of the treatment and gradually reduced thereafter (Figs. 6C and F). Intriguingly, cccDNA transcription efficiency, expressed as the molar ratio of preC mRNA over cccDNA, remained relatively stable over the eight days in untreated cells, but decreased more than 10 folds in the cells treated with IFN-α (Fig. 6G).

The results thus imply that cccDNA transcription is a primary target of IFN-α antiviral response against DHBV in the chicken hepatoma cells. The reduction of core DNA is most likely the combined effect of reduced pgRNA and inhibition of pgRNA encapsidation or accelerated degradation of pgRNA-containing nucleocapsids [51], [52]. However, the delayed reduction of cccDNA in the IFN-α-treated cells could be due to either the reduced amount of the rcDNA in the cytoplasmic nucleocapsids, the precursor of cccDNA formation [31], [53], or accelerated decay of cccDNA itself. To investigate these two possibilities, we compared the decay kinetics of DHBV DNA replication intermediates in cells treated with IFN-α and lamivudine, respectively. As shown in Fig. S4, inhibition of DHBV replication by lamivudine resulted in the decrease of core DNA and cccDNA, starting on day 2 and day 4 of the treatment, respectively. However, the amount of pgRNA in the lamivudine-treated cells only slightly reduced in comparison with the untreated cells, which could be due to the slower decay of encapsidated pgRNA compared to that of free pgRNA [44]. However, a more profound reduction of both core DNA and cccDNA in IFN-α-treated cells was observed since day 4 of the treatment, suggesting that IFN-α-induced antiviral response did not simply inhibit the cccDNA transcription and DNA replication, but also actively purged nucleocapsids and/or cccDNA.

### DHBV cccDNA transcription requires histone deacetylase activity

**Figure 7:**
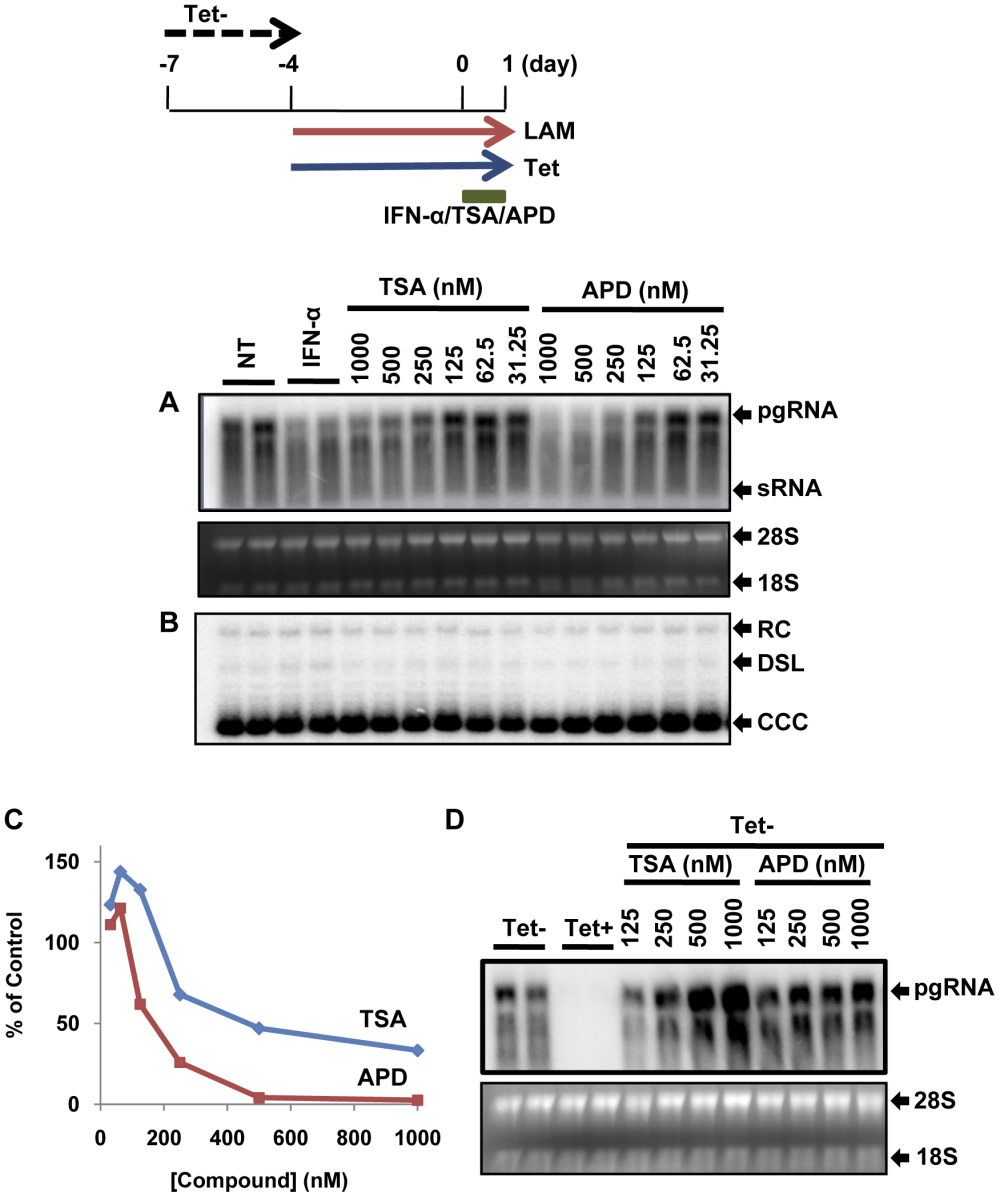
HDAC inhibitors inhibit the transcription of DHBV cccDNA, but enhance the transcription of DHBV transgene in dstet5 cells. Dstet5 cells were mock-treated or treated with IFN-α, TSA or apicidin (APD) and harvested as depicted in the top panel. DHBV mRNA (A) and cccDNA (B) were determined by Northern and Southern blot hybridization, respectively. Ribosomal RNAs served as loading controls for the Northern blot hybridization. (C) The amounts of DHBV pgRNA were quantified by phosphoimager Quantity One (Bio-Rad) and plotted as the percentage of mock-treated controls. (D) Dstet5 cells were cultured in the presence or absence of tet and mock-treated or treated with the indicated concentrations of apicidin, or TSA for 48 h. Intracellular DHBV mRNAs were analyzed by Northern blot hybridization. Ribosomal RNAs served as loading controls. pgRNA, pregenomic RNA; sRNA, mRNAs encoding envelope proteins; 28S and 18S, 28S and 18S rRNA, respectively; RC, relaxed circular DNA; DSL, double-stranded linear DNA.

To gain insight into the epigenetic regulation of cccDNA transcription and identify molecular probes helping dissect the mechanism by which IFN-α inhibits cccDNA transcription, we tested a panel of 47 small molecules that are the inhibitors or activators of DNA and histone modification enzymes. Among others, we identified histone deacetylase (HDAC) inhibitors that selectively inhibited DHBV cccDNA transcription. As illustrated in Figs. 7A to C, both Trichostatin A (TSA), a broad-spectrum HDAC inhibitor, and apicidin, a class I HDAC-specific inhibitor [54], dose-dependently reduced the amounts of pgRNA transcribed from cccDNA, but did not alter the amounts of cccDNA. The results thus suggest that DHBV cccDNA transcription may require class I HDAC activity. On the contrary, the two HDAC inhibitors enhanced the transcription of DHBV pgRNA from the viral transgene integrated in the host cellular chromosome, which is driven by a CMV-IE-tet promoter (Fig. 7D). Considering that HDAC inhibitors were shown to activate transcription of retroviral episomal circular DNA and transcription of HBV cccDNA in the unit-length linear HBV genomic DNA transfecetd HepG2 cells [38], [55], [56], our finding is rather surprising. However, the results are consistent with a previous report that n-Butyrate, an HDAC inhibitor, inhibited DHBV replication in primary duck hepatocyte cultures [57].

### HDAC inhibitors and IFN-α suppress cccDNA transcription *via* distinct mechanism

Although HDAC activity is commonly correlated with transcriptional repression, it was actually essential for the induction of many IFN-stimulated genes (ISGs) and establishment of an antiviral state [58], [59]. In order to further characterize the effect of HDAC inhibitors on cccDNA transcription, we first tested if inhibition of DHBV cccDNA transcription by HDAC inhibitors required new protein synthesis. Consistent with the results presented in Fig. 4, while the presence of CHX abolished the inhibitory effect of IFN-α on cccDNA transcription, CHX did not compromise the transcriptional suppression of DHBV cccDNA by an HDAC inhibitor, Vorinostat (suberoylanilide hydroxamic acid, SAHA) (Fig. S5). The results thus imply that unlike IFN-α that induces cellular antiviral proteins to suppress cccDNA transcription, HDAC inhibitors may directly target one or multiple pre-existing cellular proteins, such as HDACs, that are required for cccDNA transcription.

**Figure 8:**
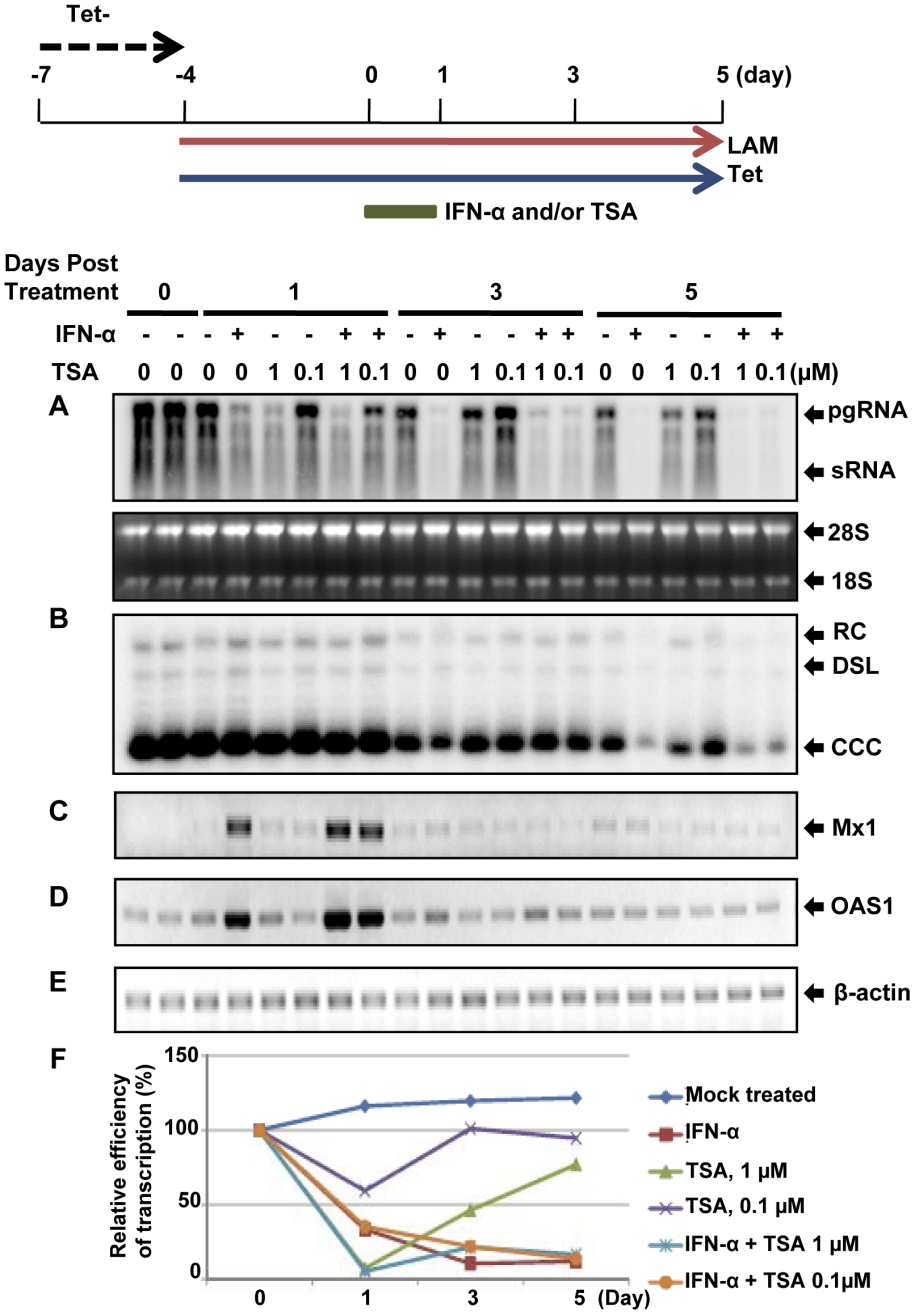
Inhibition of cccDNA transcription by IFN-α and HDAC inhibitor *via* distinct mechanisms. Dstet5 cells were treated and harvested as depicted in the top panel. DHBV mRNA (A) cccDNA (B) were determined by Northern and Southern blot hybridization, respectively. Ribosomal RNAs served as loading controls for the Northern blot hybridization. The levels of Mx1 (C), OAS1 (D) and β-actin (E) mRNA were determined by semi-quantitative RT-PCR assay. (F) Relative transcription efficiency of cccDNA was calculated as the molar ratio of pre-C mRNA over cccDNA, as determined by real-time PCR assays, and plotted as percentage of pre-treated controls. pgRNA, pregenomic RNA; sRNA, mRNAs encoding envelope proteins; 28S and 18S, 28S and 18S rRNA, respectively; RC, relaxed circular DNA; DSL, double-stranded linear DNA.

We next investigated the interaction between IFN-α and HDAC inhibitors on regulation of DHBV cccDNA function. To this end, dstet5 cells were treated with IFN-α and TSA, alone or in combination for one day and observed for additional 4 days after the cessation of the treatment. As shown in Fig. 8A, treatment of the cells with IFN-α for one day significantly reduced the amount of pgRNA and the viral RNA remained at reduced levels for an additional four days after the treatment. As observed in the previous experiments (Fig. 5B), the level of cccDNA did not change after one day of IFN-α treatment, but was reduced since the third day after the treatment (Fig. 8B). On the contrary, although treatment of the cells with TSA for one day reduced the amount of pgRNA, the level of viral RNA rebounded after the cessation of treatment. Interestingly, although TSA treatment significantly delayed the induction of ISGs, such as Mx1 and OAS1, by IFN-α (Fig. S6), it did not apparently affect the cytokine-induced reduction of viral RNA and cccDNA. To distinguish whether the prolonged reduction of pgRNA in the IFN-α-treated cells was due to the long-lasting suppression of cccDNA transcription or decrease in the amount of cccDNA, pre-C mRNA and cccDNA were quantified by real-time PCR assays and the transcription activity of cccDNA were determined as the molar ratio of the pre-C mRNA over cccDNA. As shown in Fig. 8F, while cccDNA transcription activity gradually recovered after the cessation of TSA treatment, IFN-α treatment for 24 h induced a prolonged suppression of cccDNA transcription, which was apparently unaffected by the TSA treatment.

Taken together, the results presented herein clearly demonstrated that IFN-α and HDAC inhibitors suppressed DHBV cccDNA transcription with different characteristics and thus *via* distinct mechanisms. Specifically, while HADC inhibitors promptly inhibited cccDNA transcription by directly targeting cellular functions required for cccDNA transcription, IFN-α induced a long-lasting suppression of cccDNA transcription through induction of cellular antiviral protein synthesis. In addition, HDAC inhibitors did not apparently affect the stability of cccDNA, but IFN-α induced an accelerated decay of cccDNA.

### IFN-α treatment does not induce cccDNA methylation, but reduces acetylation of DHBV cccDNA minichromosome-associated histone H3

Epigenetic modification of DNA and histones has been demonstrated to play important roles in gene transcription regulation [60], [61]. The prolonged suppression of cccDNA transcription by IFN-α evokes a hypothesis that the cytokine may induce certain epigenetic modifications of cccDNA minichromosomes to sustain the inhibition. To test this hypothesis, we first determined whether or not IFN-α treatment altered cccDNA methylation. As depicted in Fig. S7, sequence analysis of DHBV genome (GeneBank accession number K01834.1) identified three GC-rich islands spanning nucleotide 278 to 407, 1038–1232 and 1559 to 1733, respectively. Bisulfate sequence analysis of the three GC-rich islands and DHBV core promoter region (nt 2172 to 2529) revealed that cccDNA were unmethylated in the untreated dstet5 cells and that IFN-α treatment for two days did not induce cccDNA methylation. The result thus suggests that DNA methylation does not play an essential role in IFN-α suppression of cccDNA transcription.

**Figure 9:**
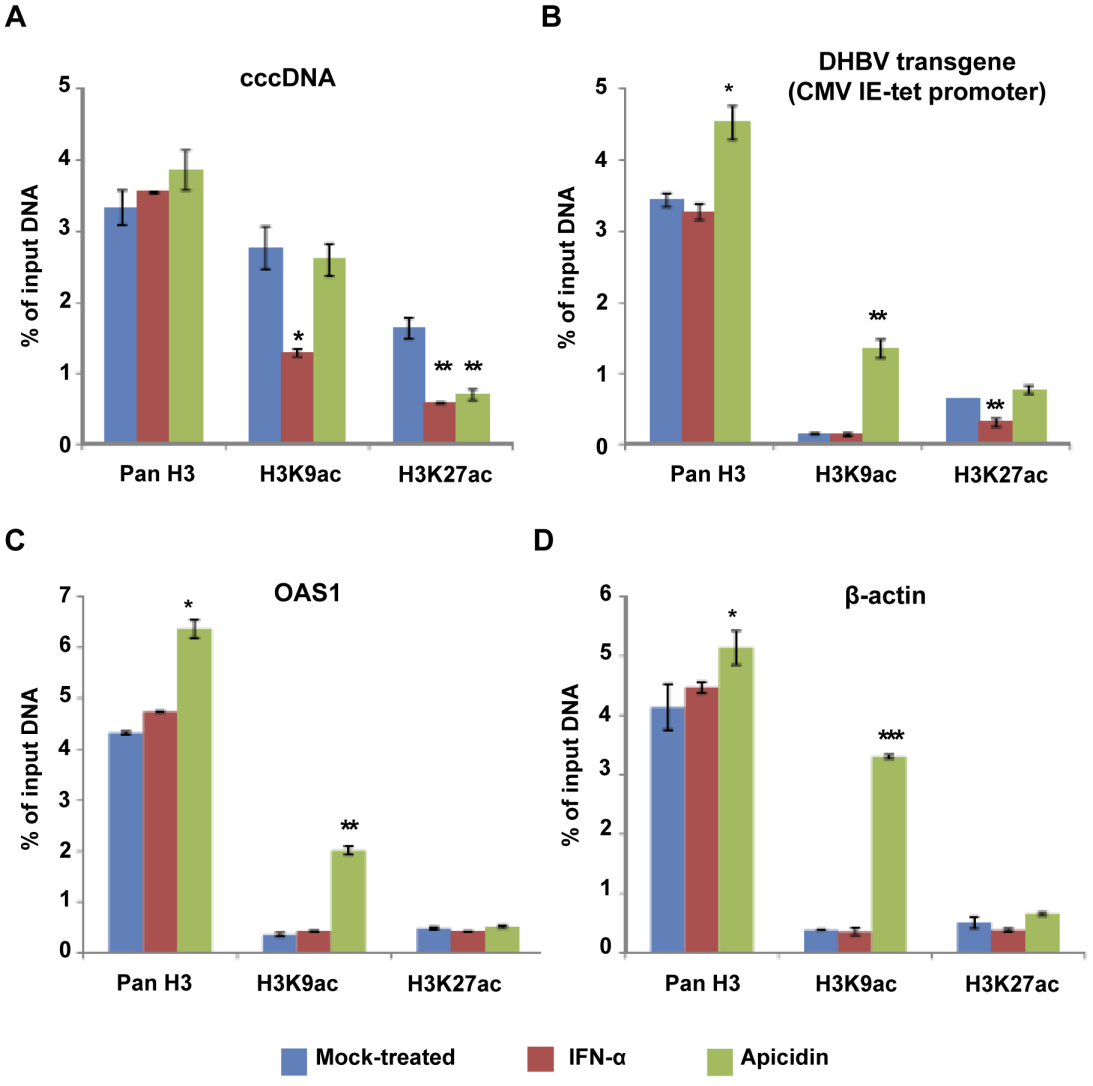
ChIP analysis of histone modifications in DHBV cccDNA minichromosomes. Dstet5 cells were cultured in the absence of tet for three days and then cultured with media containing tet and lamivudine (10 µM) for additional 4 days. Cells were then mock-treated or treated with 100 U/ml of IFN-α or 1 µM of apicidin (APD) for 24 h. ChIP were carried out with antibodies specific for histone H3, H3K9ac and H3K27ac, respectively. Rabbit IgG was used as a negative control to evaluate the non-specific binding. Quantitative PCR assays were performed with primers specific to cccDNA (A), DHBV transgene (B), OAS1 (C) or β-actin (D). Results were presented as percentages of input DNA and are the mean values and standard derivations of a representative triplicate experiment. *, ** and *** indicate P<0.05, 0.01 and 0.001 in comparison with mock-treated controls, respectively.

Previous studies suggest that hyperacetylation of histone H3 is correlated with a high transcriptional activity of HBV cccDNA in the livers of HBV-infected patients [38]. In particular, epigenetic modifications of histone 3 lysine 9 (H3K9) and 27 (H3K27) have been shown to play important roles in regulating the expression of a variety of host and viral genes [62], [63]. Accordingly, we tested the acetylation status of H3K9 and H3K27 in cccDNA minichromosomes, promoter regions of the DHBV transgene, OAS1 and β-actin genes in the dstet5 cells mock-treated or treated with IFN-α or apicidin. As shown in Fig. 9A, consistent with the high transcriptional activity of cccDNA, the cccDNA-associated H3K9 and H3K27 were hyper-acetylated in mock-treated cells. Interestingly, treatment of the cells with IFN-α significantly reduced the acetylation levels of the cccDNA-associated H3K9 and H3K27. However, apicidin treatment only significantly reduced the acetylation levels of the cccDNA-associated H3K27, but not H3K9. In marked contrast, the basal levels of H3K9 and H3K27 acetylation were much lower in the promoter regions of the DHBV transgene, OAS1 and β-actin genes than that in the cccDNA minichrosmosomes. While apicidin treatment significantly increased the acetylation of H3K9, but not H3K27, in the promoter regions of all the three genes, IFN-α treatment only significantly reduced the H3K27 acetylation in the CMV-tet promoter region of DHBV transgene (Figs. 9B to D).

In addition, methylation of H3K9 and H3K27 has been demonstrated to be correlated with heterochromatin formation and polycomb repressive complex-mediated gene repression, respectively [64], [65]. In order to determine if IFN-α induced gene silencing or heterochromatin formation of cccDNA, we tested the abundance of trimethylated H3K9 (H3K9me3) and dimethylated H3K27 (H3K27me2)-associated with cccDNA minichromosomes by ChIP analysis. Consistent with the actively transcriptional status of cccDNA and other three genes under the investigation, only very low levels (less than 0.1%) of H3K9me3 and H3K27me2 were detected in cccDNA minichromosomes and the promoter regions of HBV transgene, OAS1 and β-actin (Fig. S8). Interestingly, neither IFN-α nor apicidin treatment increased the abundance of the H3K9me3 and H3K27me2 in cccDNA and other three tested promoters.

In summary, the results presented in this section demonstrated that IFN-α suppression of DHBV cccDNA transcription was associated with the reduced acetylation levels of H3K9 and H3K27 in cccDNA minichromosomes. However, the low levels of H3K9me3 and H3K27me2 in cccDNA minichromosomes strongly suggest that the suppression of DHBV cccDNA transcription by either IFN-α or apicidin is most likely not due to polycomb repressive complex-mediated gene repression or heterochromatin formation of cccDNA minichromosomes.

## Discussion

Among the many important aims of future HBV research is the development of therapies of finite duration capable of eradicating HBV infection. Accomplishment of this goal requires elimination and/or cure of all the HBV-infected cells in the body. In fact, more than 90% of adulthood HBV infections are resolved by host immune responses *via* a coordinated kill and cure of infected cells [15], [66], [67]. While the virus specific cytolytic T lymphocytes are considered to be the primary force to kill the infected hepatocytes, the cure of the infected hepatocytes is believed to be achieved by the inflammatory cytokine-induced cellular responses [13]–[15]. Considering that cccDNA is the most stable replication intermediate of hepadnaviral DNA, elimination or transcriptional silencing of cccDNA in the infected hepatocytes is essential for the cytokines to cure the HBV-infected cells. Unfortunately, due to the lack of convenient cell culture systems for efficient infection [31], [32], the effects of the cytokines on the stability and transcriptional activity of the cccDNA have not been extensively investigated. In recent years, Levrero and colleagues studied HBV transcription in the linear full-length HBV DNA-transfected human hepatoma cells, in which cccDNA molecules were presumably formed by circularization of input linear HBV DNA [38], [39]. Using this assay, this research group established a role for HBx protein in cccDNA minichromosome transcription and investigated the mechanism by which IFN-α suppresses HBV cccDNA transcription [18], [39]. However, due to the transient nature of the assay, the long-term effects of the cytokines on cccDNA transcription and maintenance could not be investigated. Using the chicken hepatoma cell-based assay established herein, we investigated the effects and underlying mechanism of IFN-α on DHBV cccDNA transcription and maintenance and revealed the following unique features of hepadnavirus cccDNA metabolism and transcription regulation.

### IFN-α induces a prompt and long-lasting suppression of cccDNA transcription

While the prompt inhibition of cccDNA transcription by IFN-α requires antiviral protein synthesis, the prolonged suppression of cccDNA transcription implies that the cytokine may induce a profound alteration of cccDNA minichromosomes, such as formation of a heterochromatin-like structure, to silence the transcription [63], [68], [69]. DNA methylation and histone modifications have been shown to play important roles in regulating the expression of a variety of viral genes and the establishment of latency [62], [63], [70]. In particular, previous studies suggested that increased CpG methylation of cccDNA was associated with low serum HBV DNA levels and hyperacetylation of histone H3 was correlated with a high transcription activity of HBV cccDNA in the livers of chronic hepatitis B patients [71], [72] [38]. Our studies reported herein further supported this notion, by demonstrating that IFN-α treatment significantly reduced the acetylation levels of cccDNA-associated H3K9 and H3K27 (Fig. 9A).

However, the failure to induce H3K9me3 and H3K27me2 in DHBV cccDNA minichromosomes by IFN-α implies that IFN-α suppression of DHBV cccDNA transcription is most likely not due to polycomb repressive complex-mediated gene repression or heterochromatin formation of cccDNA minichromosomes. Hence, further understanding of the nature, spatial distribution and dynamics of cccDNA epigenetic modifications in response to IFN and other inflammatory cytokines and their relationship with transcription suppression and elimination of the episomes are essential to understand how the host immune system noncytolytically controls HBV infection.

### IFN-α induces a delayed response that accelerates the decay of cccDNA

cccDNA can only be synthesized from relaxed circular (rc) or double-stranded linear (dsl) DNA in the incoming or newly formed nucleocapsids in the cytoplasm, but cannot replicate themselves through semiconservative replication in the nuclei [8], [9]. In principle, elimination of the nuclear cccDNA could take place *via* either selective destruction of the minichromosomes by cellular nucleases or dilution and unequal partition into daughter cells during cell division [73], [74]. It is conceivable that IFN and/or other cytokines may activate cellular response to epigenetically modify cccDNA minichromosomes, which marks the episomes for selective decay in stationary cells or prevents the minichromosomes to be re-enclosed into nuclei after mitosis and subsequently degraded by cytoplasmic nucleases. Alternatively, the epigenetic modification could also alter cccDNA partitioning into daughter cells. Considering the fact that the accelerated decline of cccDNA in IFN-α-treated cells could be observed when viral DNA replication was arrested by lamivudine treatment (Fig. 5B) and was significantly faster than that occurred in the cells treated with lamivudine (Fig. S4C), we favor the hypothesis that IFN-induced antiviral response actively purges cccDNA. However, the nature of epigenetic modifications associated with the cccDNA decay and the role of cell division in the cytokine-induced cccDNA elimination remain to be determined.

### DHBV cccDNA transcription requires HDAC activity

HDAC activity is commonly correlated with transcriptional repression and establishment of latent viral infection [54], [75]. Accordingly, HDAC inhibitors have been employed to activate HIV transcription in the latently infected cells and eradicate latent HIV infection [76]. However, a recent microarray study revealed that treatment of cells with HDAC inhibitors affected the expression of a large fraction of cellular genes and the expression of approximately 50% of the affected genes were inhibited by the treatment [77]. In agreement with the previous reports [58], [59], IFN-αinduced expression of ISGs, such as of Mx1 and OAS1, in the chicken hepatoma cells required HDAC activity (Fig. S6). In this study, we demonstrated for the first time that DHBV cccDNA transcription could be inhibited by multiple compounds that inhibit HDAC activity. Unlike IFN-α, inhibition of cccDNA transcription by HDAC inhibitors did not require new protein synthesis and cccDNA transcription gradually resumed upon removal of the inhibitors. The results thus suggested that HDAC inhibitors might directly target one or multiple pre-existing cellular proteins essential for cccDNA transcription. Moreover, the observed hyperacetylation of cccDNA associated histone H3 and requirement of HDAC activity for cccDNA transcription suggest that the dynamic acetylation and deacetylation of cccDNA associated histones might be essential for cccDNA transcription. Hence, identification of HDAC(s) and histone acetylases (HATs) involved in the dynamic processes not only should advance our understanding of cccDNA transcription regulation, but also provide potential therapeutic targets for selective inhibition of cccDNA transcription.

Ironically, our observation that HDAC inhibitors inhibited DHBV cccDNA transcription is contradictory with a previous report showing that TSA increased the HBV cccDNA transcription in the unit-length HBV genomic DNA-transfected HepG2 cells [38]. To resolve this discrepancy, we took the advantage of a recent discovery that expression of human sodium taurocholate cotransporting polypeptide (NTCP) in HepG2 cells conferred susceptibility of HBV infection and investigated the effects of HDAC inhibitors on HBV gene expression in HBV infected HepG2 cells [78]. As shown in Fig. S9, treatment of HBV infected cells at 24 h post infection with apicidin dose-dependently reduced the numbers of HepG2/NTCP cells that expressed HBcAg antigen and decreased the amounts of 3.5 kb HBV mRNA and secreted HBeAg. Similar results were obtained with TSA treatment (data not shown). Although the mechanism remains to be further investigated, the results do suggest that a cellular function sensitive to the HDAC inhibitors is required for HBV RNA transcription and protein expression.

Finally, the requirement of HDAC activity for DHBV cccDNA transcription seems to mechanistically conflict with the observed association of reduced H3K9 and H3K27 acetylation and IFN-α suppression of DHBV cccDNA transcription. Because HDAC inhibitors did not apparently affect the long-lasting suppression of IFN-α on cccDNA transcription and IFN-α-induced decay of DHBV cccDNA (Fig. 8), it is thus possible that the reduced acetylation of H3H9 and H3K27 in DHBV cccDNA minichromosomes by IFN-αis catalyzed by HDACs that are not sensitive to the conventional HDAC inhibitors, such as sirtuins. Alternatively, it is also possible that the reduced acetylation of H3K9 and H3K27 in DHBV cccDNA minichromosomes by IFN-α is due to the disruption of a dynamic acetylation and deacetylation of histone H3 through preventing the recruitment of histone acetyltransferases (HATs) into the minichromosomes. Hence, further investigations on the recruitment of individual HDACs, sirtuins and HATs to cccDNA minichromosomes and a comprehensive analysis of the nature, spatial distribution and dynamics of cccDNA-associated histone acetylation are essential to ultimately clarify the role of histone acetylation in cccDNA metabolism and transcription regulation.

In conclusion, we have established a convenient cell culture system harboring abundant and actively transcribing DHBV cccDNA produced from its authentic precursor, the cytoplasmic nucleocapsid DNA. This assay system allows for the investigation of hepadnaviral cccDNA metabolism and transcription regulation without the interference of the large amount of transfected viral DNA. Although there are differences in many aspects of replication cycle between DHBV and HBV, it is conceivable that as investigation of viral DNA synthesis and other replication steps, the principles uncovered in the DHBV model system on cccDNA metabolism and regulation should provide insight on understanding HBV cccDNA biology and clues for the development of therapeutics to control chronic hepatitis B.

## Materials and Methods

### Cell lines, IFN-α and chemicals

Dstet5 is a chicken hepatoma cell (LMH)-derived stable cell line supporting the replication of an envelope protein-deficient DHBV genome in a tetracycline dependent manner [44]. Dstet5 cells were maintained in DMEM/F12 medium (Mediatech) supplemented with 10% fetal bovine serum, 100 U/ml penicillin, 100 µg/ml streptomycin, 1 µg/ml tetracycline and 200 µg/ml G-418. Chicken IFN-α was produced and titrated as described previously [44]. HepG2-derived cell line expressing Sodium taurocholate cotransporting polypeptide (NTCP) (HepG2/NTCP) was established and maintained as described previously [78]. Trichostatin A, apicidin and Vorinostat were purchased from ENZO Life Sciences. Antibodies against histone H3-pan (Cat. No. 07-690), Acetyl-histone H3 (Lys27) (Cat. No. 07-360), Acetyl histone H3 (Lys9) (Cat. No. ABE18), trimethyl-Histone H3 (lys 9) (Cat. No. 07-442) and dimethyl-Histone H3 (lys 27) (Cat. No. 07-421) were purchased from Millipore. Normal Rabbit IgG (Cat. No. 2729) was purchased from Cell Signaling, Inc.

### DHBV DNA and RNA analyses

Intracellular DHBV core DNA was extracted as described previously [44], [53]. One half of the DNA sample from each well of 12-well plates was resolved by electrophoresis into a 1.5% agarose gel and transferred onto Hybond-XL membrane.

Extraction of protein-free DHBV DNA was carried out by using a modified Hirt extraction procedure [79], [80]. Briefly, cells from one 35 mm diameter dish were lysed in 3 ml of 10 mM Tris-HCl (pH 7.5), 10 mM EDTA and 0.7% SDS. After 5 minutes incubation at room temperature, the lysate was mixed with 1 ml of 2.5M KCl and incubated at room temperature for 30 min and followed by centrifugation at 10,000 g for 15 min at 4°C. The supernatants were extracted twice with phenol, and once with phenol∶chloroform. DNA was precipitated with two volumes of ethanol overnight at room temperature and dissolved in TE buffer (10 mM Tris-HCl, pH 8.0, 1 mM EDTA). One half of the protein-free DNA sample from each well of 12-well plates was then resolved in a 1.5% agarose gel and transferred onto Hybond-XL membrane.

For viral RNA analysis, total cellular RNA was extracted with TRIzol reagents (Life Technologies). Five micrograms of total RNA was resolved in 1.5% agarose gel containing 2.2 M formadelhyde and transferred onto Hybond-XL membrane in 20× SSC buffer and blotted onto Hybond-XL membrane.

For the detection of viral DNA and RNA, membranes were probed with either an α-^32^P-UTP (800 Ci/mmol, Perkin Elmer) labeled minus or plus strand specific full-length DHBV riboprobe. Hybridization was carried out in 5 ml EKONO hybridization buffer (G-Biosciences, St. Louis, MO) with 1 hour pre-hybridization at 65°C and overnight hybridization at 65°C, followed by a 1 hour wash with 0.1X SSC and 0.1% SDS at 68°C. The membrane was exposed to a phosphoimager screen and hybridization signals were scanned and quantified with QuantityOne software (Bio-Rad, Hercules, CA).

### PCR

Expression of chicken IFN-stimulated genes, Mx1 and OAS1, and a house-keeping gene β-actin was quantified by semi-quantitative RT-PCR. Total cellular RNA was extracted with TRIzol and cDNA was synthesized with oligo-(dT)_12–18_ primer and Superscript III DNA polymerase (Invitrogen) by following the manufacturer's direction. The PCRs were carried out in a 25-µl reaction mixture with the Advantage cDNA PCR kit (Clontech). The PCR annealing temperatures selected varied depending on the primers selected for amplification. The prime sequences are listed in Table S1.

Mx1, OAS1, ACTB and DHBV preC mRNA were also quantitatively detected with SuperScript III Platinum One-Step qRT-PCR Kit (Invitrogen). Forty nanograms of total cellular RNA was reverse transcribed and amplified in a 20-µl reaction mixture per reaction. One step qPCR was carried out as follows. cDNA was synthesized at 60°C for 10 minutes and denatured at 95°C for 2 minutes, followed immediately by 40 cycles of amplification: 95°C, 15 seconds; 59°C, 35 seconds. For preC mRNA measurement, because its amplicon is larger than 250 bp, the maximum the protocol recommended, its amplification condition was adjusted as the follows: 40 cycles of amplification: 95°C, 15 seconds; 60°C, 65 seconds. DHBV core DNA and cccDNA is measured by LightCycler 480 SYBR Green I Master PCR kit (Invitrogen). The PCR was carried out as follows: denatured at 95°C for 5 minutes, followed by 40 cycles of amplification: 95°C, 15 seconds; 60°C, 30 seconds. The amounts of DHBV core DNA and cccDNA were calculated based on a standard curve with known amounts of DHBV DNA under the same amplification conditions.

### Bisulfite sequencing

Dstet5 cells were cultured in the absence of tet for three days and in the presence of 1 µg/ml tet and 10 µM lamivudine for additional 4 days. The cells were then mock-treated or treated with 100 U/ml IFN-αfor 48 h. DHBV cccDNA was extracted with an alkali denaturing method described before [44] and modified using the EZ DNA Methylation-Gold Kit (ZymoResearch, Orange, CA) following the manufacturer's protocol. The sequence of each sample was determined using Chromas Lite 2.33 (Technelysium Pty Ltd).

### ChIP assay

ChIP assay was performed with an EpiTect Chip One-Day Kit (Qiagen) by following the procedures provided by the manufacturer with slight modifications. Briefly, Dstet5 cells were cultured in the absence of tet for three days and in the presence of 1 µg/ml tet and 10 µM lamivudine for additional 4 days. The cells were then mock-treated or treated with 100 U/ml IFN-αfor 48 h. Cells were harvested by trypsinization and pelleted by centrifugation at 800 g for 10 min. The cells were suspended in PBS at a density of 3×10^6^ cells/ml and fixed in 1% formaldehyde at room temperature for 10 minutes. The fixed cells were pelleted at 800 g for 10 minutes at 4°C and resuspended by addition of immunoprecipitation lysis buffer supplemented with proteinase inhibitor cocktail at a density of 1×10^7^ cells/ml. Five hundred microliters of the cell lysates were sonicated by cup horn (Sonicator XL2020, Misonix) at a setting of 10W for 2 minutes on and 2 minutes off. The sonication was repeated once at the same setting. This sonication condition has been showed steadily breaking cellular DNA into 500–800 bp fragments. For pre-clear, immunoprecipitation and DNA extraction, we strictly followed the instruction provided in the EpiTect ChIP One-Day Kit (Qiagen). The obtained DNA was subjected to quantitative analysis by real-time PCR with primers specified in the Table S1.

### HBV infection of HepG2/NTCP cells and apicidin treatment

HepG2/NTCP cells in 48-well plate were incubated with HBV virions at a multiplicity of infection of 100 for 24 h. Subsequently, the cells were washed three times with culture medium and incubated with the indicated concentrations of apicidin for 8 h (Figure S9). The culture medium was then replaced with fresh medium and changed every other day. Cells were stained on day 7 post infection to detect HBV core antigen (HBcAg) by an indirect immunofluorescent assay [78]. Secreted HBeAg was measured by ELISA [78]. Intracellular HBV 3.5 kb RNA was quantified with a quantitative RT-PCR [78].

## Supporting Information

Figure S1 **IFN-α dose-dependently reduces the amount of DHBV mRNAs transcribed from cccDNA.** Dstet5 cells were treated and harvested as depicted in the top panel. DHBV mRNA (A), core DNA (B) and cccDNA (C) were determined by Northern and Southern blot hybridization, respectively. Ribosomal RNAs served as loading controls for the Northern blot hybridization. The amount of DHBV pgRNA were quantified by phosphoimager Quantity One (Bio-Rad), the relative amount of pgRNA was presented with the mock-treated cells set as 100% (panel A). pgRNA, pregenomic RNA; sRNA, mRNAs encoding envelope proteins; 28S and 18S, 28S and 18S rRNA, respectively; RC, relaxed circular DNA; DSL, double-stranded linear DNA; SS, single stranded DNA.
(TIF)

Figure S2 **IFN-α does not accelerate the decay of DHBV mRNA.** (A) Dstet5 cells were cultured in tet-free medium containing 10 µM lamivudine for three days to allow the accumulation of viral mRNAs. The cells were then mock-treated or treated with 100 U/ml IFN-α for the indicated periods of time. Intracellular DHBV mRNAs were analyzed by Northern blot hybridization. Ribosomal RNAs served as loading controls. (B) The amount of DHBV pgRNA was quantified by phosphoimager Quantity One (Bio-Rad) and plotted as percentage of the pre-treatment control. pgRNA, pregenomic RNA; sRNA, mRNAs encoding envelope proteins.
(TIF)

Figure S3 **A time course study of IFN-α inhibition on DHBV cccDNA transcription.** Dstet5 cells were treated and harvested as depicted in the top panel. DHBV mRNA (A) and cccDNA (B) were determined by Northern and Southern blot hybridization, respectively. Ribosomal RNAs served as loading controls for the Northern blot hybridization. The amount of DHBV pgRNA was quantified by phosphoimager Quantity One (Bio-Rad) and presented as percentage of pre-treatment controls. pgRNA, pregenomic RNA; sRNA, mRNAs encoding envelope proteins; 28S and 18S, 28S and 18S rRNA, respectively; RC, relaxed circular DNA; DSL, double-stranded linear DNA.
(TIF)

Figure S4 **Comparative study of IFN-α and lamivudine on DHBV replication.** Dstet5 cells were left untreated or treated with IFN-α (100 U/ml) or lamivudine (LAM, 10 µM) and harvested as depicted in the top panel. DHBV mRNA (A), core DNA (B) and cccDNA (C) were determined by Northern and Southern blot hybridization, respectively. Ribosomal RNAs served as loading controls for the Northern blot hybridization. The amounts of DHBV pgRNA, core DNA and cccDNA were quantified by phosphoimager Quantity One (Bio-Rad) and presented as percentage of a pre-treatment control. pgRNA, pregenomic RNA; sRNA, mRNAs encoding envelope proteins; 28S and 18S, 28S and 18S rRNA, respectively; RC, relaxed circular DNA; DSL, double-stranded linear DNA; SS, single stranded DNA.
(TIF)

Figure S5 **Inhibition of cccDNA transcription by SAHA does not require protein synthesis.** Dstet5 cells were cultured in the absence of tet for 5 days and followed by culturing in the presence of 1 µg/ml tet for another three weeks. (A) The cells were then left untreated or treated with the indicated concentration of SAHA or IFN-α (100 U/ml) for 24 h. Viral RNA was detected by Northern blot hybridization. Ribosomal RNA served as loading controls. (B) The cells were mock-treated or treated with IFN-α (100 U/ml), SAHA (25 µM), CHX (10 µg/ml), alone or in combination, for 6, 9, 12 and 15 h, respectively. DHBV mRNA (upper panel) cccDNA (lower panel) were determined by Northern and Southern blot hybridization, respectively. Ribosomal RNAs served as loading controls for the Northern blot hybridization (middle panel). pgRNA, pregenomic RNA; sRNA, mRNAs encoding envelope proteins; 28S and 18S, 28S and 18S rRNA, respectively; RC, relaxed circular DNA; DSL, double-stranded linear DNA.
(TIF)

Figure S6 **Effects of TSA on IFN-α-induced ISG expression.** Dstet5 cells were left untreated or treated with 100 U/ml IFN-α and/or 1 µM TSA for the indicated periods of time. The levels of Mx1, OAS1 and β-actin mRNA were determined by real-time PCR assays. Results were presented as fold of induction in comparison with untreated controls.
(TIF)

Figure S7 **IFN-α does not induce cccDNA methylation.** (A) DHBV minimal core promoter (CP, nt 2410–2529), Enhancer (En, nt 2172–2350) and three predicted CpG islands located at nt 278–407, 1038–1232 and 1559–1733 are depicted. (B) Alignment of the parent and predicted bisulfate DNA sequence of unmethylated CpG island I and the bisulfate sequences of the corresponding region of cccDNA prepared from dstet5 cells in the absence (NT) or presence of 100 U/ml IFN-α for 2 days. (C and D) The raw sequence data of the DHBV cccDNA CpG island I are presented.
(TIF)

Figure S8 **ChIP analysis of histone 3 methylation in DHBV cccDNA minichromosomes.** Treatment of Dstet5 cells is described in Materials and Methods. ChIP was carried out with antibodies specific for histone H3, H3K9me3 and H3K27me2, respectively. Rabbit IgG was used as a negative control to evaluate the non-specific binding. Quantitative PCR assays were performed with primers specific to cccDNA (A), DHBV transgene (B), OAS1 (C) or β-actin (D). Results were presented as percentages of input DNA and are the mean values and standard derivations of a representative triplicate experiment.
(TIF)

Figure S9 **Apicidin inhibits the expression of HBcAg and HBeAg in NTCP-expressing HepG2 cells infected by HBV.** HBcAg in HepG2/NTCP cells on day 7 after infection were visualized by immunofluorescent staining with a monoclonal antibody against HBV core protein (A) and relative percentage of HBcAg-positive cells in the infected cultures were plotted (B). The amounts of secreted HBeAg at the indicated times post infection were determined by ELISA and presented as the percent of mock-treated controls (C). The amounts of 3.5 kb HBV mRNA in mock-treated (Ctrl) and 0.5 µM apicidin treated cells at day 6 after HBV infection were quantified with a qRT-PCR assay and expressed as copies per nanogram of total cellular RNA (D). Mean values and standard derivations of a representative triplicate experiment are presented.
(TIF)

Table S1 **Sequences of primers used in the study.**
(DOCX)
