# Supplementary figures and images for: Alpha-Interferon Suppresses Hepadnavirus Transcription by Altering Epigenetic Modification of cccDNA Minichromosomes
Source: PLoS Pathog. 2013 Sep 12;9(9):e1003613. doi: 10.1371/journal.ppat.1003613 (PMC3771898; doi:10.1371/journal.ppat.1003613)

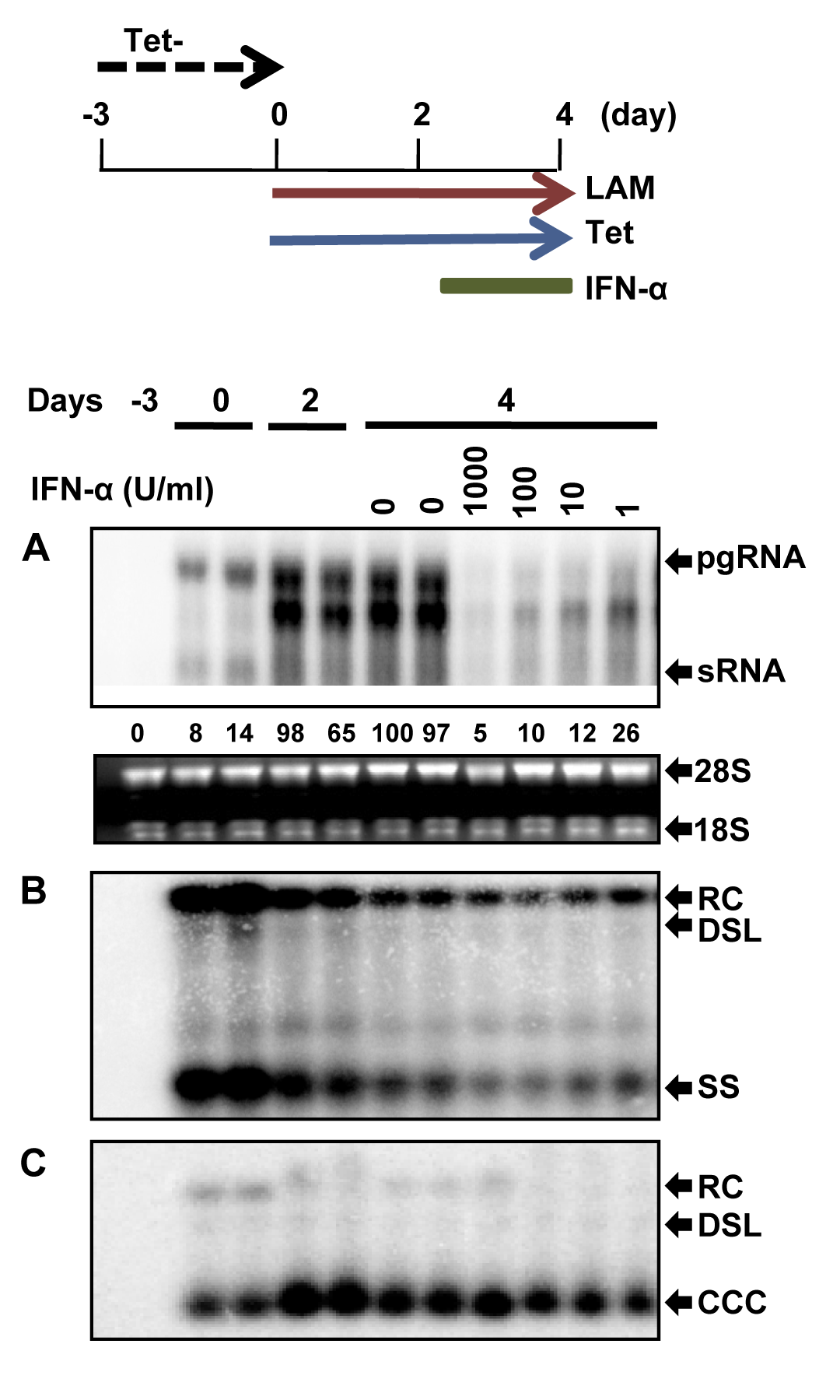

Supplement: Figure S1 — IFN-α dose-dependently reduces the amount of DHBV mRNAs transcribed from cccDNA. Dstet5 cells were treated and harvested as depicted in the top panel. DHBV mRNA (A), core DNA (B) and cccDNA (C) were determined by Northern and Southern blot hybridization, respectively. Ribosomal RNAs served as loading controls for the Northern blot hybridization. The amount of DHBV pgRNA were quantified by phosphoimager Quantity One (Bio-Rad), the relative amount of pgRNA was presented with the mock-treated cells set as 100% (panel A). pgRNA, pregenomic RNA; sRNA, mRNAs encoding envelope proteins; 28S and 18S, 28S and 18S rRNA, respectively; RC, relaxed circular DNA; DSL, double-stranded linear DNA; SS, single stranded DNA. (TIF) [file ppat.1003613.s001.tif]

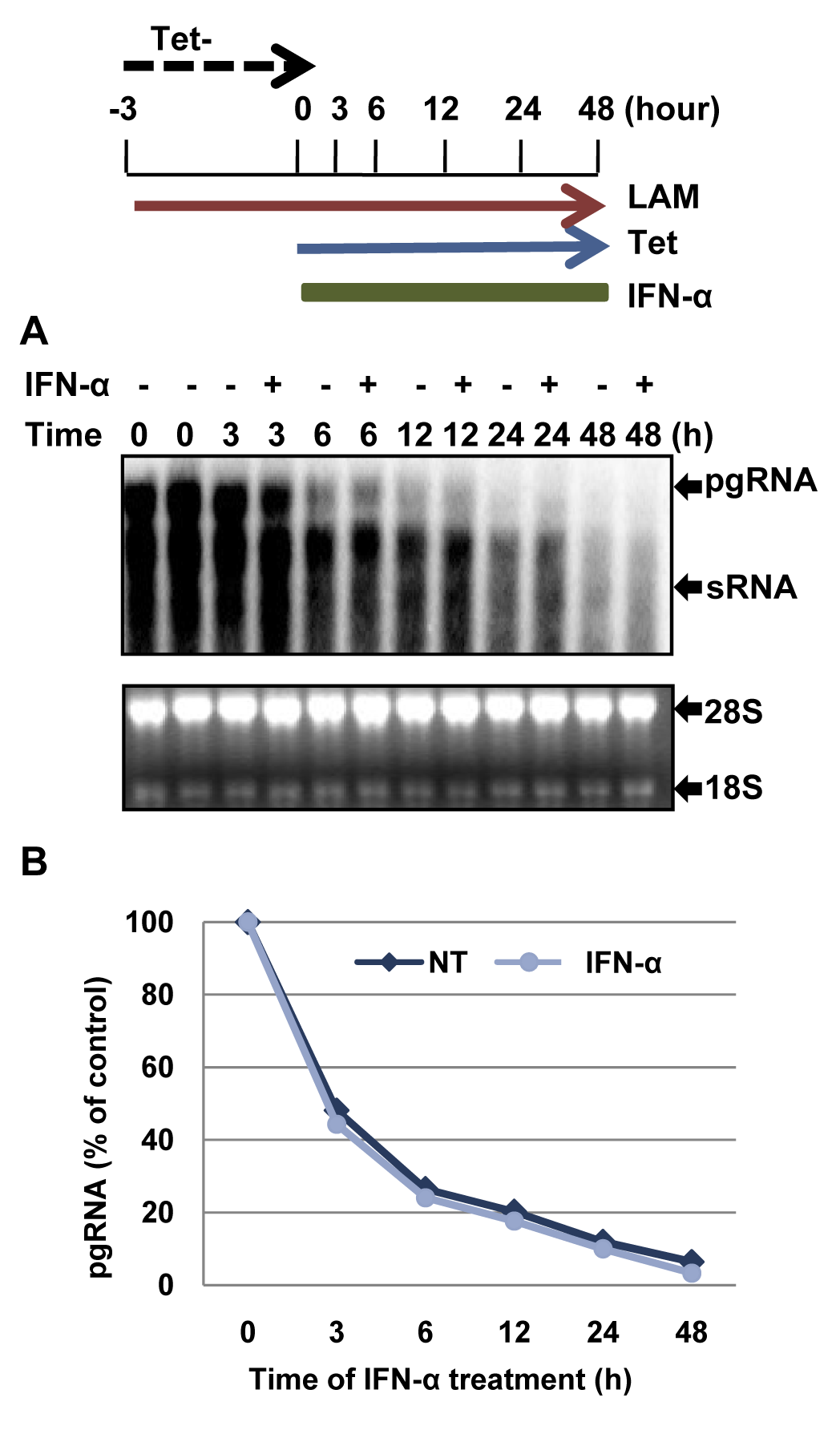

Supplement: Figure S2 — IFN-α does not accelerate the decay of DHBV mRNA. (A) Dstet5 cells were cultured in tet-free medium containing 10 µM lamivudine for three days to allow the accumulation of viral mRNAs. The cells were then mock-treated or treated with 100 U/ml IFN-α for the indicated periods of time. Intracellular DHBV mRNAs were analyzed by Northern blot hybridization. Ribosomal RNAs served as loading controls. (B) The amount of DHBV pgRNA was quantified by phosphoimager Quantity One (Bio-Rad) and plotted as percentage of the pre-treatment control. pgRNA, pregenomic RNA; sRNA, mRNAs encoding envelope proteins. (TIF) [file ppat.1003613.s002.tif]

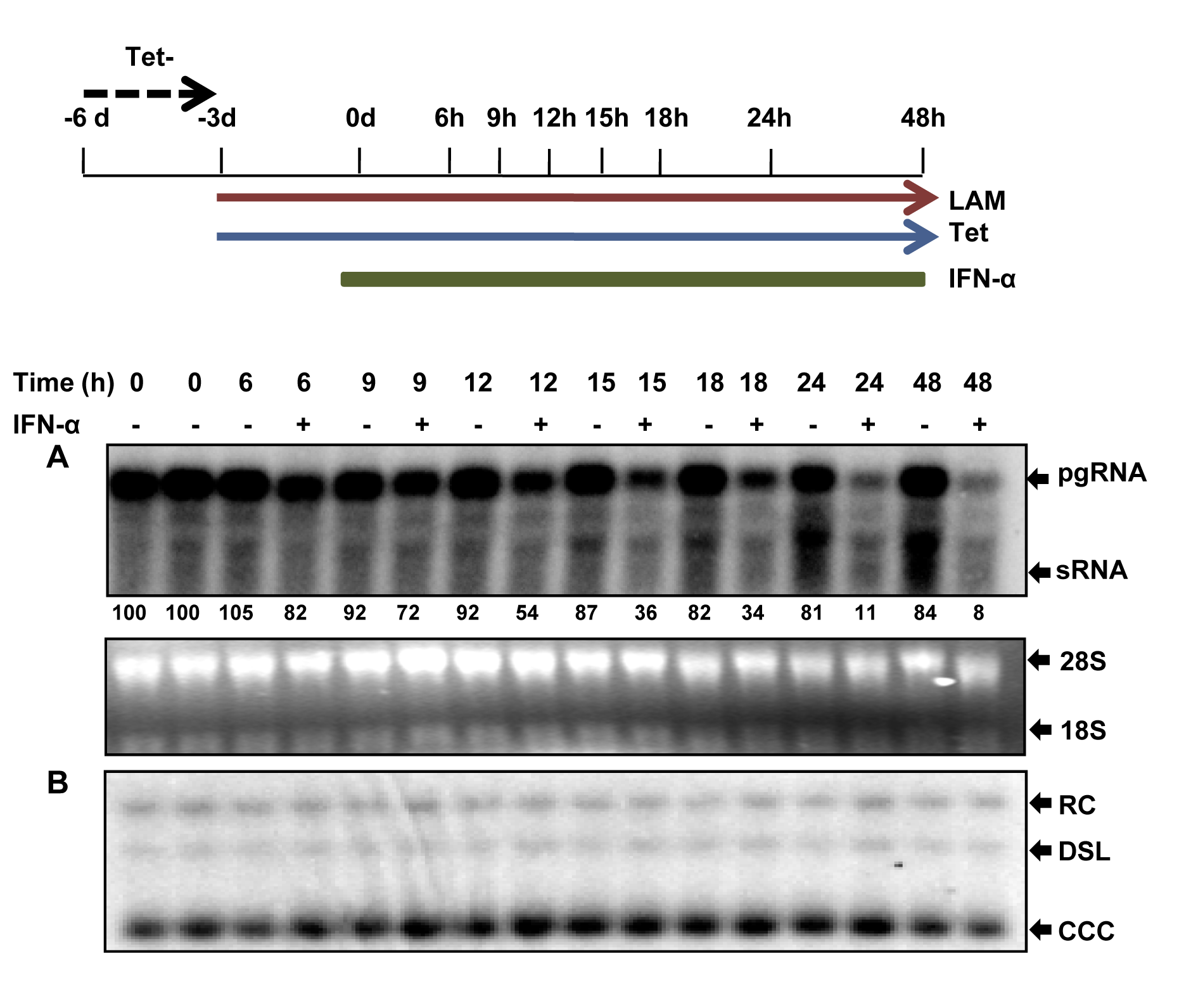

Supplement: Figure S3 — A time course study of IFN-α inhibition on DHBV cccDNA transcription. Dstet5 cells were treated and harvested as depicted in the top panel. DHBV mRNA (A) and cccDNA (B) were determined by Northern and Southern blot hybridization, respectively. Ribosomal RNAs served as loading controls for the Northern blot hybridization. The amount of DHBV pgRNA was quantified by phosphoimager Quantity One (Bio-Rad) and presented as percentage of pre-treatment controls. pgRNA, pregenomic RNA; sRNA, mRNAs encoding envelope proteins; 28S and 18S, 28S and 18S rRNA, respectively; RC, relaxed circular DNA; DSL, double-stranded linear DNA. (TIF) [file ppat.1003613.s003.tif]

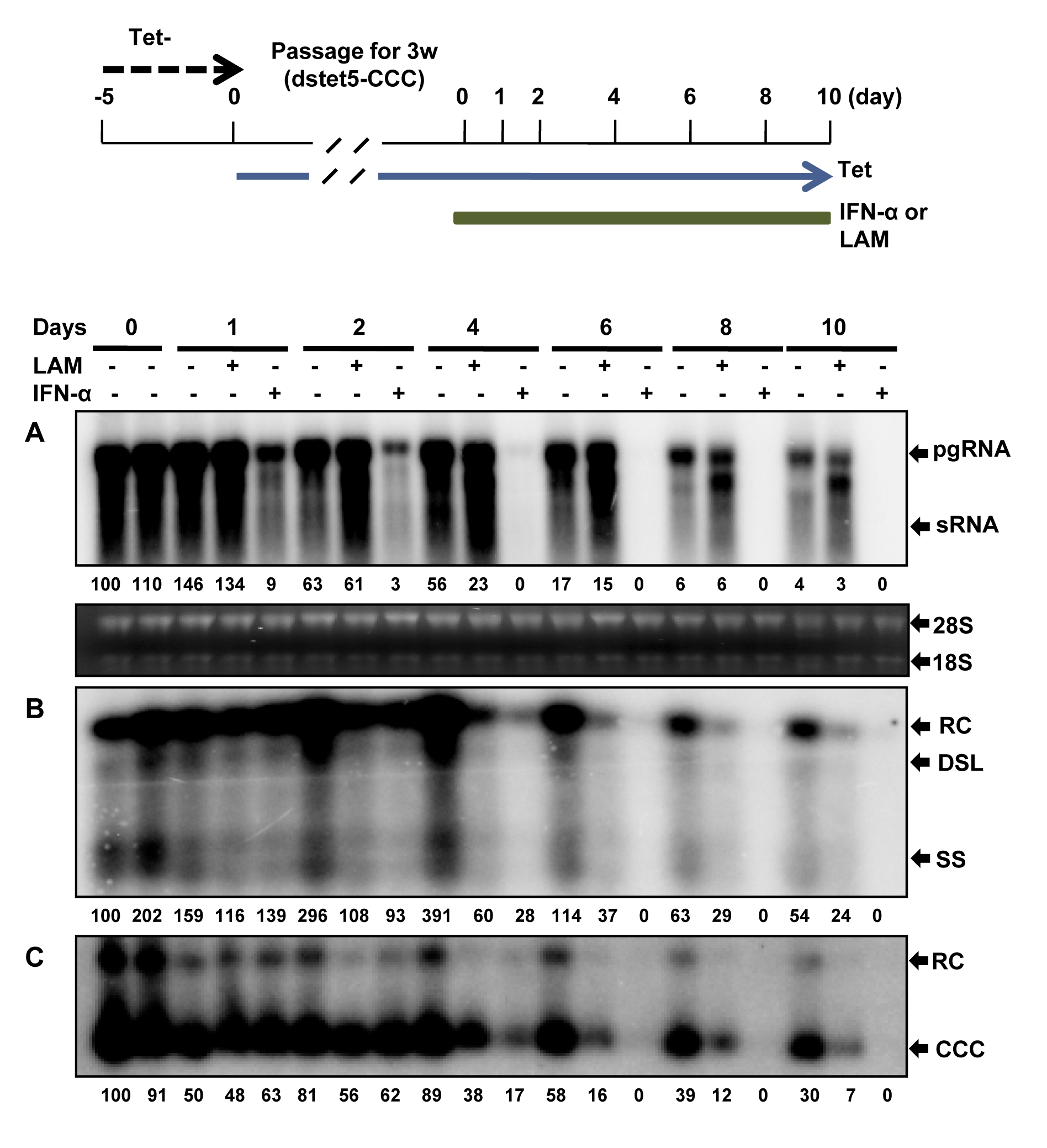

Supplement: Figure S4 — Comparative study of IFN-α and lamivudine on DHBV replication. Dstet5 cells were left untreated or treated with IFN-α (100 U/ml) or lamivudine (LAM, 10 µM) and harvested as depicted in the top panel. DHBV mRNA (A), core DNA (B) and cccDNA (C) were determined by Northern and Southern blot hybridization, respectively. Ribosomal RNAs served as loading controls for the Northern blot hybridization. The amounts of DHBV pgRNA, core DNA and cccDNA were quantified by phosphoimager Quantity One (Bio-Rad) and presented as percentage of a pre-treatment control. pgRNA, pregenomic RNA; sRNA, mRNAs encoding envelope proteins; 28S and 18S, 28S and 18S rRNA, respectively; RC, relaxed circular DNA; DSL, double-stranded linear DNA; SS, single stranded DNA. (TIF) [file ppat.1003613.s004.tif]

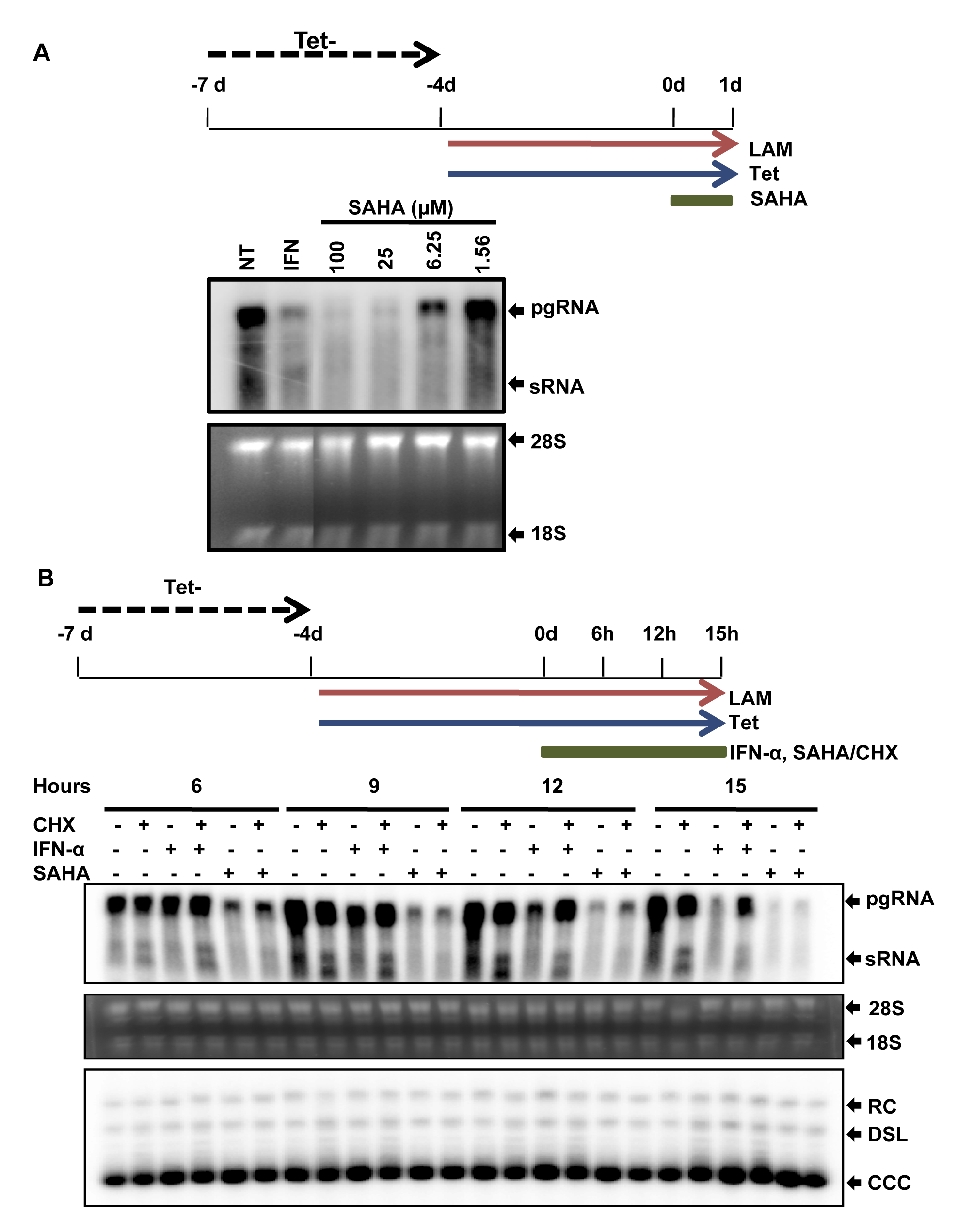

Supplement: Figure S5 — Inhibition of cccDNA transcription by SAHA does not require protein synthesis. Dstet5 cells were cultured in the absence of tet for 5 days and followed by culturing in the presence of 1 µg/ml tet for another three weeks. (A) The cells were then left untreated or treated with the indicated concentration of SAHA or IFN-α (100 U/ml) for 24 h. Viral RNA was detected by Northern blot hybridization. Ribosomal RNA served as loading controls. (B) The cells were mock-treated or treated with IFN-α (100 U/ml), SAHA (25 µM), CHX (10 µg/ml), alone or in combination, for 6, 9, 12 and 15 h, respectively. DHBV mRNA (upper panel) cccDNA (lower panel) were determined by Northern and Southern blot hybridization, respectively. Ribosomal RNAs served as loading controls for the Northern blot hybridization (middle panel). pgRNA, pregenomic RNA; sRNA, mRNAs encoding envelope proteins; 28S and 18S, 28S and 18S rRNA, respectively; RC, relaxed circular DNA; DSL, double-stranded linear DNA. (TIF) [file ppat.1003613.s005.tif]

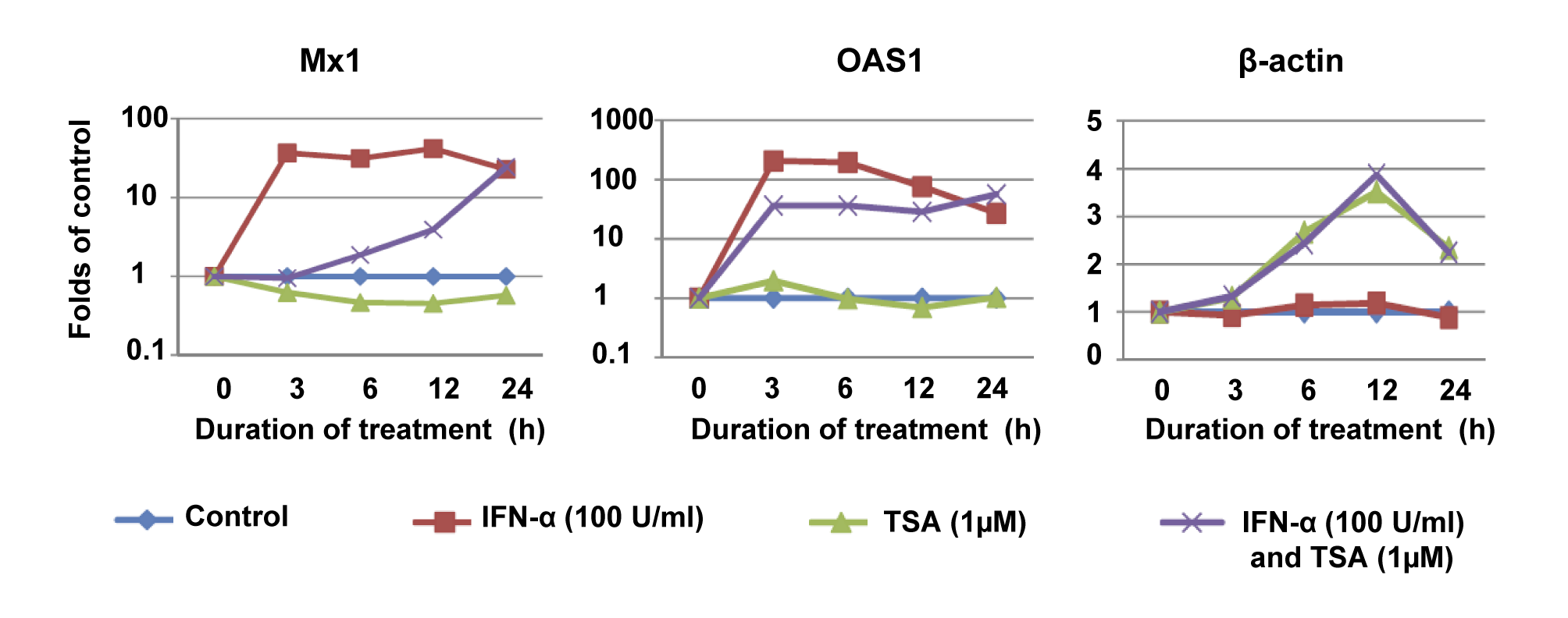

Supplement: Figure S6 — Effects of TSA on IFN-α-induced ISG expression. Dstet5 cells were left untreated or treated with 100 U/ml IFN-α and/or 1 µM TSA for the indicated periods of time. The levels of Mx1, OAS1 and β-actin mRNA were determined by real-time PCR assays. Results were presented as fold of induction in comparison with untreated controls. (TIF) [file ppat.1003613.s006.tif]

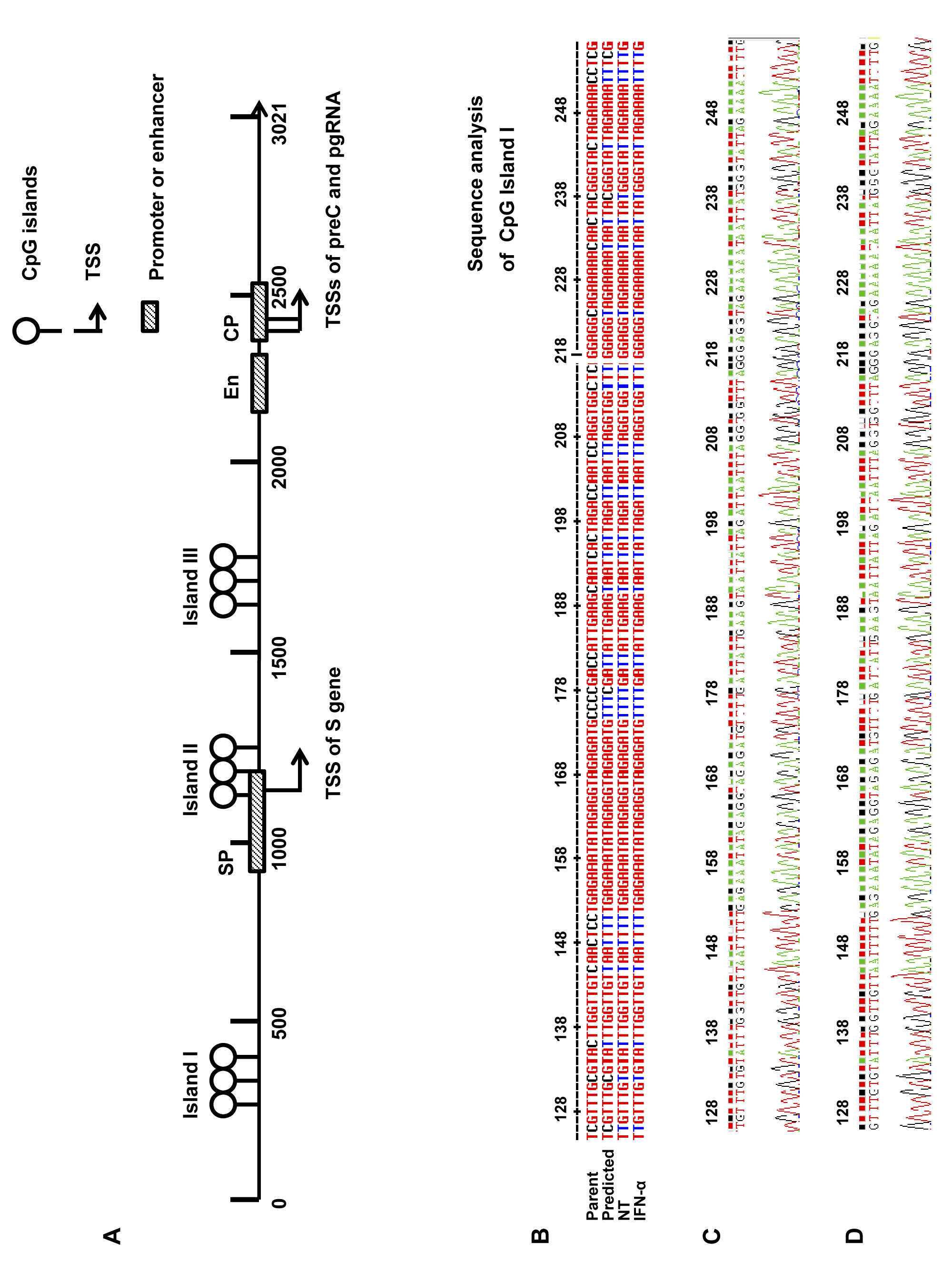

Supplement: Figure S7 — IFN-α does not induce cccDNA methylation. (A) DHBV minimal core promoter (CP, nt 2410–2529), Enhancer (En, nt 2172–2350) and three predicted CpG islands located at nt 278–407, 1038–1232 and 1559–1733 are depicted. (B) Alignment of the parent and predicted bisulfate DNA sequence of unmethylated CpG island I and the bisulfate sequences of the corresponding region of cccDNA prepared from dstet5 cells in the absence (NT) or presence of 100 U/ml IFN-α for 2 days. (C and D) The raw sequence data of the DHBV cccDNA CpG island I are presented. (TIF) [file ppat.1003613.s007.tif]

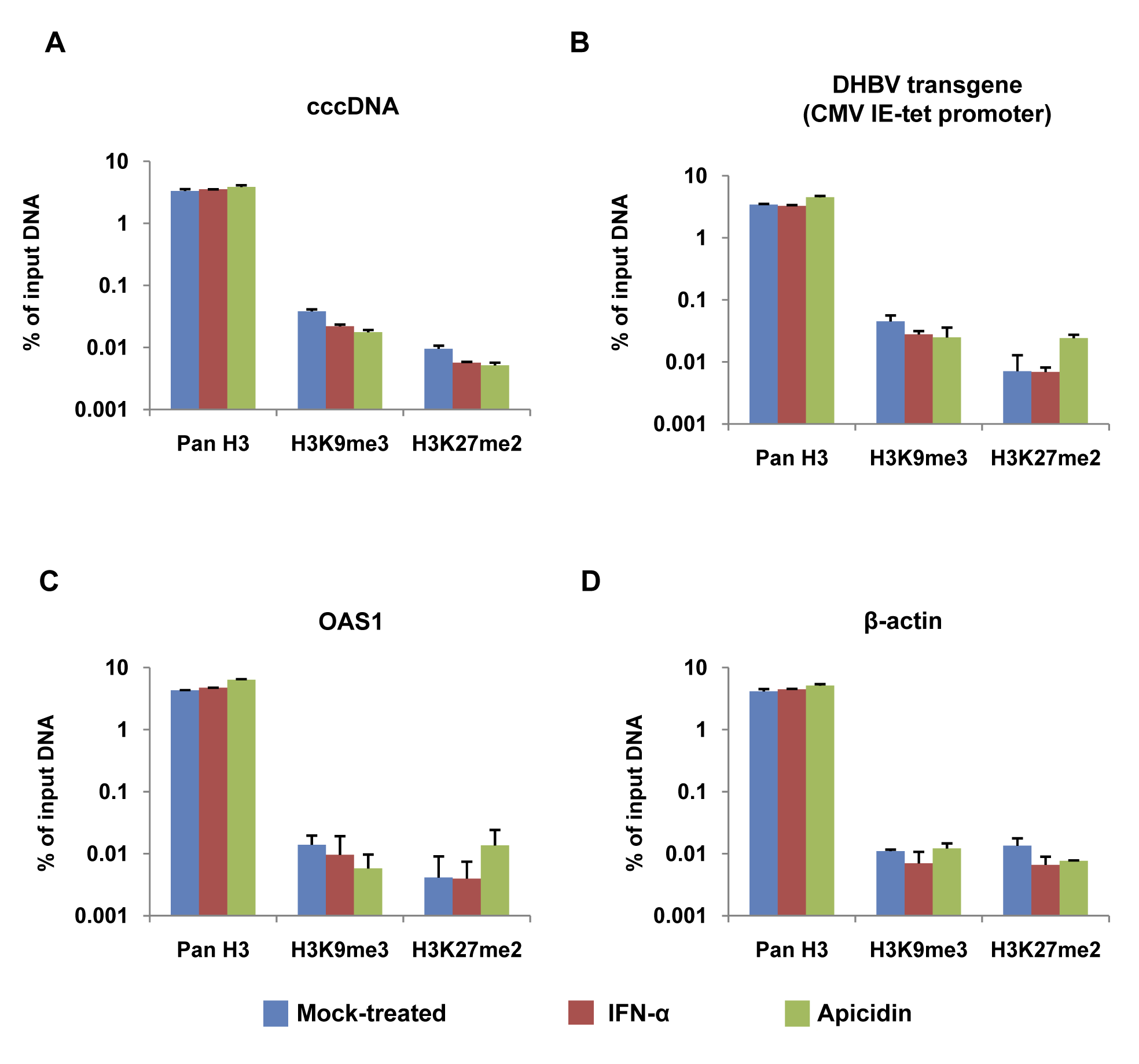

Supplement: Figure S8 — ChIP analysis of histone 3 methylation in DHBV cccDNA minichromosomes. Treatment of Dstet5 cells is described in Materials and Methods. ChIP was carried out with antibodies specific for histone H3, H3K9me3 and H3K27me2, respectively. Rabbit IgG was used as a negative control to evaluate the non-specific binding. Quantitative PCR assays were performed with primers specific to cccDNA (A), DHBV transgene (B), OAS1 (C) or β-actin (D). Results were presented as percentages of input DNA and are the mean values and standard derivations of a representative triplicate experiment. (TIF) [file ppat.1003613.s008.tif]

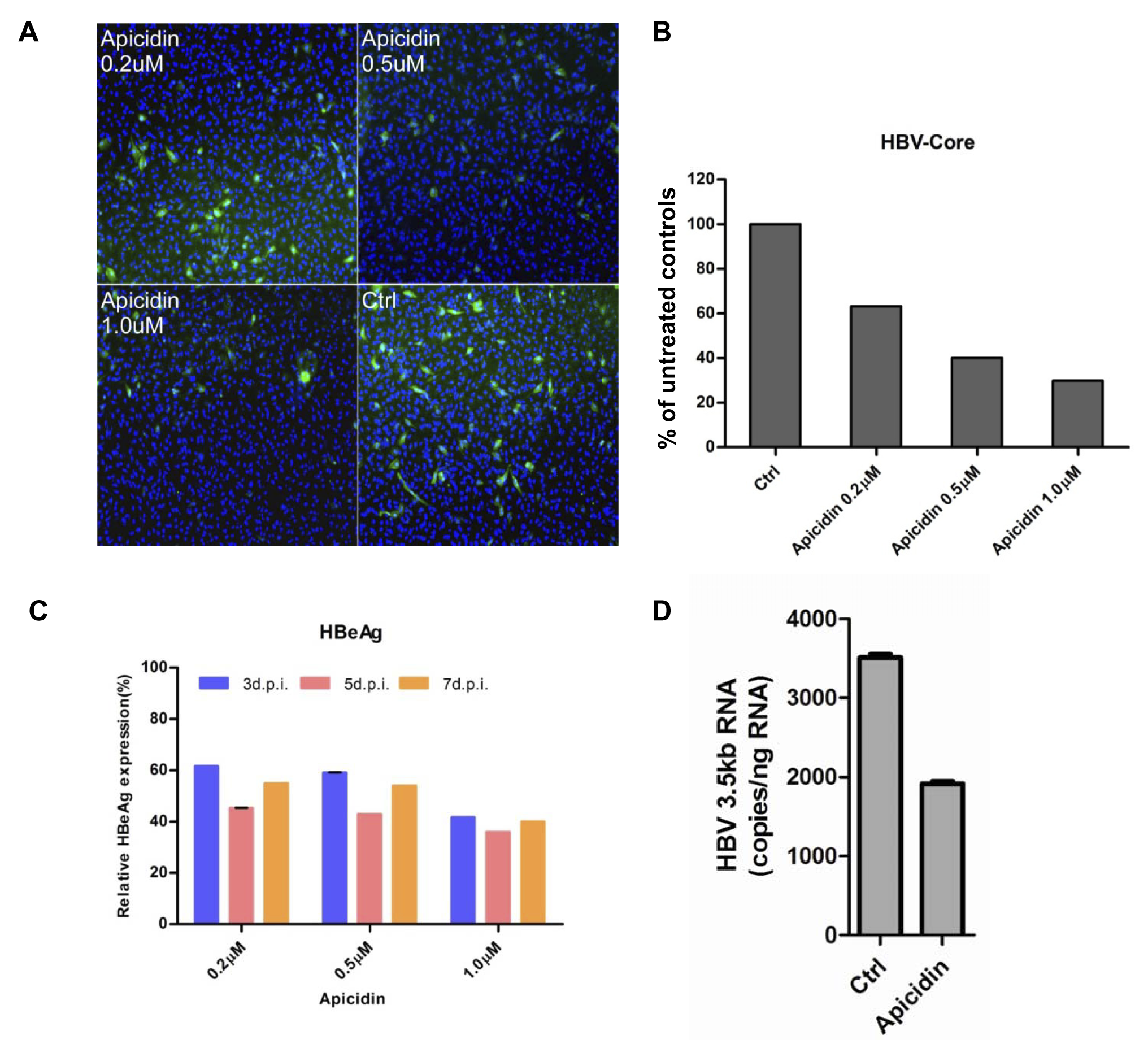

Supplement: Figure S9 — Apicidin inhibits the expression of HBcAg and HBeAg in NTCP-expressing HepG2 cells infected by HBV. HBcAg in HepG2/NTCP cells on day 7 after infection were visualized by immunofluorescent staining with a monoclonal antibody against HBV core protein (A) and relative percentage of HBcAg-positive cells in the infected cultures were plotted (B). The amounts of secreted HBeAg at the indicated times post infection were determined by ELISA and presented as the percent of mock-treated controls (C). The amounts of 3.5 kb HBV mRNA in mock-treated (Ctrl) and 0.5 µM apicidin treated cells at day 6 after HBV infection were quantified with a qRT-PCR assay and expressed as copies per nanogram of total cellular RNA (D). Mean values and standard derivations of a representative triplicate experiment are presented. (TIF) [file ppat.1003613.s009.tif]
