# Supplementary material for: Alpha-Interferon Suppresses Hepadnavirus Transcription by Altering Epigenetic Modification of cccDNA Minichromosomes
Source: PLoS Pathog. 2013 Sep 12;9(9):e1003613. doi: 10.1371/journal.ppat.1003613 (PMC3771898; doi:10.1371/journal.ppat.1003613)
Supplement: Table S1 — Sequences of primers used in the study. (DOCX) [file ppat.1003613.s010.docx]

**Table S1. Sequences of primers used in the study**

| Gene | Experiment | sequences |
| --- | --- | --- |
| Mx1 | RT-PCR | Forward: TACGAAGCTGGAGGAGCCAGC  Reverse: TACCAGGTATTGGTAGGCTTTGTTGAG |
| OASA | RT-PCR | Forward: TGA GGT GGG AGA TGG GGT T  Reverse: CACCTTCACAGGTCGGGTG |
| ACTB | RT-PCR | Forward: ATGAAGCCCAGAGCAAAAGAG  Reverse: TCGAAATCCAGTGCGACGTAG |
| Mx1 | qRT-PCR | Forward: CTTTGTCTGGTGTTGCTCTTC  Reverse: GCTGTATTTCTGTGTTGCGG |
| OASA | qRT-PCR | Forward: CATCCTGCCTGCTTACGAC  Reverse: GGCCAACAGCTTCACGTAG |
| ACTB | qRT-PCR | Forward: ACCCCAAAGCCAACAGAG  Reverse: CCAGAGTCCATCACAATACCAG |
| preC mRNA | qRT-PCR | Forward: TGATTGGACGGCTTTTCCATAC  Reverse: GCCCTGTGTAGTCTGCCAGAAG |
| Core DNA | qPCR | Forward: AATTGTACTTTGTCCCGAGCA  Reverse: AGGAGGTTTGTGCCTGGAT |
| cccDNA | qPCR | Forward: TGATTGGACGGCTTTTCCATAC  Reverse: ACACGACAACAGCAATGTAGACG |
| cccDNA | ChIP assay | Forward: TGATTGGACGGCTTTTCCATAC  Reverse: ACACGACAACAGCAATGTAGACG |
| Transgene | ChIP assay | Forward: GGTAGGCGTGTACGGTGGG  Reverse: GTCACACACGACAACAGCAATG |
| OASA | ChIP assay | Forward: CACCCTCCCATCACCTTATC  Reverse: CGTGATGGCGACTTCCTAA |
| ACTB | ChIP assay | Forward: ATTCGGCTGCCTTGACCT  Reverse: AGCAAGCGCTGAGTAAGGAG |
